# Too much of a good thing: Adaption to iron (II) intoxication in *Escherichia coli*

**DOI:** 10.1093/emph/eoaa051

**Published:** 2021-01-18

**Authors:** Misty D Thomas, Akamu J Ewunkem, Sada Boyd, Danielle K Williams, Adiya Moore, Kristen L Rhinehardt, Emma Van Beveren, Bobi Yang, Anna Tapia, Jian Han, Scott H Harrison, Joseph L Graves

**Affiliations:** Department of Biology, North Carolina Agricultural and Technical State University, 1601 E. Market St, Greensboro, NC 27411, USA; BEACON, Center for the Study of Evolution in Action, Michigan State University, East Lansing, MI 48824, USA; BEACON, Center for the Study of Evolution in Action, Michigan State University, East Lansing, MI 48824, USA; Department of Nanoengineering, Joint School of Nanoscience and Nanoengineering, North Carolina Agricultural and Technical State University and UNC Greensboro, 2907 E. Gate City Blvd., Greensboro, NC 27401, USA; Department of Nanoengineering, Joint School of Nanoscience and Nanoengineering, North Carolina Agricultural and Technical State University and UNC Greensboro, 2907 E. Gate City Blvd., Greensboro, NC 27401, USA; Department of Biology, North Carolina Agricultural and Technical State University, 1601 E. Market St, Greensboro, NC 27411, USA; Department of Biology, North Carolina Agricultural and Technical State University, 1601 E. Market St, Greensboro, NC 27411, USA; Computational Data Science and Engineering, North Carolina Agricultural and Technical State University, 1601 E. Market Street, Greensboro, NC 27411, USA; Department of Nanoengineering, Joint School of Nanoscience and Nanoengineering, North Carolina Agricultural and Technical State University and UNC Greensboro, 2907 E. Gate City Blvd., Greensboro, NC 27401, USA; Department of Nanoengineering, Joint School of Nanoscience and Nanoengineering, North Carolina Agricultural and Technical State University and UNC Greensboro, 2907 E. Gate City Blvd., Greensboro, NC 27401, USA; Department of Nanoengineering, Joint School of Nanoscience and Nanoengineering, North Carolina Agricultural and Technical State University and UNC Greensboro, 2907 E. Gate City Blvd., Greensboro, NC 27401, USA; Department of Biology, North Carolina Agricultural and Technical State University, 1601 E. Market St, Greensboro, NC 27411, USA; BEACON, Center for the Study of Evolution in Action, Michigan State University, East Lansing, MI 48824, USA; Department of Biology, North Carolina Agricultural and Technical State University, 1601 E. Market St, Greensboro, NC 27411, USA; BEACON, Center for the Study of Evolution in Action, Michigan State University, East Lansing, MI 48824, USA; Department of Biology, North Carolina Agricultural and Technical State University, 1601 E. Market St, Greensboro, NC 27411, USA; BEACON, Center for the Study of Evolution in Action, Michigan State University, East Lansing, MI 48824, USA

**Keywords:** iron resistance, *E. coli*, experimental evolution, pleiotropy, gene expression

## Abstract

**Background:**

There has been an increased usage of metallic antimicrobial materials to control pathogenic and multi-drug resistant bacteria. Yet, there is a corresponding need to know if this usage leads to genetic adaptations that could produce more harmful strains.

**Methodology:**

Experimental evolution was used to adapt *Escherichia coli* K-12 MG1655 to excess iron (II) with subsequent genomic analysis. Phenotypic assays and gene expression studies were conducted to demonstrate pleiotropic effects associated with this adaptation and to elucidate potential cellular responses.

**Results:**

After 200 days of adaptation, populations cultured in excess iron (II), showed a significant increase in 24-h optical densities compared to controls. Furthermore, these populations showed increased resistance toward other metals [iron (III) and gallium (III)] and to traditional antibiotics (bacitracin, rifampin, chloramphenicol and sulfanilamide). Genomic analysis identified selective sweeps in three genes; *fecA*, *ptsP* and *ilvG* unique to the iron (II) resistant populations, and gene expression studies demonstrated that their cellular response may be to downregulate genes involved in iron transport (*cirA* and *fecA*) while increasing the oxidative stress response (*oxyR, soxS* and *soxR*) prior to FeSO_4_ exposure.

**Conclusions and implications:**

Together, this indicates that the selected populations can quickly adapt to stressful levels of iron (II). This study is unique in that it demonstrates that *E. coli* can adapt to environments that contain excess levels of an essential micronutrient while also demonstrating the genomic foundations of the response and the pleiotropic consequences. The fact that adaptation to excess iron also causes increases in general antibiotic resistance is a serious concern.

**Lay summary:** The evolution of iron resistance in *E. coli* leads to multi-drug and general metal resistance through the acquisition of mutations in three genes (*fecA, ptsP* and *ilvG*) while also initiating cellular defenses as part of their normal growth process.

## BACKGROUND AND OBJECTIVE

Metal ions are essential for many bacterial processes, but in excess, can serve as antibacterials. As a result, both limitation and intoxication-based mechanisms have been used by hosts as effective strategies in limiting the growth of microbial pathogens [1, 2]. To date, studies of the mechanisms by which bacteria evolve adaptation to metal limitation have been common, and have shown that microorganisms use strategies including; enzymatic transformation (redox and methylation) and upregulation of metal-binding proteins for increased storage and sequestration [3, 4]. While much less is understood about adaptation toward metal intoxication, it is known that both essential physiological processes of bacteria rely upon a delicate balance between efflux and influx in order to maintain both an appropriate cellular quota and the kinetically accessible, labile pool of micronutrients [1].

Despite poorly understood mechanisms, and the various constitutive strategies bacteria use to counteract damaging effects, both limitation and intoxication most often lead to growth arrest and ultimately death [1, 3–5]. However, *de novo* mutation rates in bacteria are sufficient to allow for the evolution of resistance to a variety of substances including metals. Thus, genes conferring resistance to toxic metal ions are widespread among bacteria, allowing them to first acclimate and then eventually adapt to the excess metal, provided that the bacteria have been able to first survive via some interim acclimation to these conditions of stress [1].

Iron being one of the most important biologically relevant micronutrients is a versatile prosthetic component serving as a co-factor for proteins involved in many essential cellular processes including nitrogen fixation, metabolism and respiration [6]. Despite its critical role in bacterial metabolism, acquiring iron is one of the greatest challenges for bacterial growth [7, 8] and as a result, iron deficiency is one of the most common scenarios of nutritional stresses [9]. Furthermore, under aerobic condition, iron can be extremely toxic due to the generation of harmful reactive oxygen species (ROS) through the Fenton/Haber–Weiss reactions which produces superoxide ($O_{2}^{-}$), hydrogen peroxide (H_2_O_2_) and hydroxyl radicals (^•^OH) [10]. These ROS damage the cell through processes of [Fe-S] cluster destructions, protein carbonylation, oxidation of Cys/Met residues, lipid peroxidation and DNA damage [11, 12]. Finally, increased oxidative stress often leads to an increase in mismetallation thereby further inactivating essential enzymes [5].

Global iron homeostasis is primarily controlled by the transcriptional regulator Fur [13], which controls expression of about 90 coding and non-coding RNAs and as a result, plays a role in repressing oxidative stress, acid resistance and virulence and is the principal regulator of iron transport [14]. In aerobic environments, iron predominantly occurs as ferric iron (Fe^3+^), however, Fe (OH)_3_ is poorly soluble in aqueous solution (as low as 1 0^−18^ M at pH 7.0) making acquisition more difficult. Therefore, in order to uptake iron from the environment, bacteria have evolved sophisticated acquisition systems including both low- and high-affinity transporters that can import iron in either its free state or chelated in siderophores [15]. Mechanisms of iron export are less well known but it has been shown that in some cases, both enterobactin production and iron-citrate efflux help to confer oxidative stress resistance [16]. As aerobic bacteria are continuously exposed to ROS through normal metabolic processes, they have evolved mechanisms to counteract their toxic effects. This includes the use of iron storage proteins (ferritins and bacterioferritins), which sequester iron, making it unavailable for Fenton chemistry [17, 18] and enzymes that can degrade ROS (i.e. Sod, KatA, AhpCF and KatG) some themselves requiring iron as a co-factor [19]. Due to the co-dependency between iron homeostasis and the oxidative-stress response, it is apparent that there is significant regulatory cross-talk between these two systems [10, 20].

Both ionic iron and iron nanoparticles are being proposed as methods for controlling multi-drug resistant bacteria, [21–23] as they have even been shown to enhance the activity of traditional antibiotics [24–26]. For example, against *Escherichia coli,* Fe^2+^-loaded chitosan nanoparticles have an minimum inhibitory concentration (MIC) of 10 mg/ml with even better activity against gram-positive bacteria and have been furthermore proposed to be used as a food preservative [27]. T808hereby, their continued use will increase the exposure of microorganisms to high levels of iron. It is, therefore, important to understand if bacteria can adapt to survive in excess iron, and if so, to pre-emptively elucidate the cellular response that will ensue. We are using *E. coli* as a model to begin to understand how gram-negative bacteria evolve in environments containing stressful levels of iron and evaluate the genomic and cellular consequences of this adaptation. This work is the beginning point for understanding the mechanisms of both iron intoxication and iron adaptation as will be essential before the deployment of iron-based compounds as methods of microbial control.

## METHODOLOGY

### Experimental evolution

*Escherichia coli* K-12 MG1655 (ATCC #47076) was chosen for this study and all growth experiments were performed in Davis Minimal Broth (DMB; Difco, Sparks, MD) supplemented with 10% dextrose as the carbon source and 0.3 µM thiamine hydrochloride. All cultures were grown at 37°C with shaking at 115 rpm. To begin, the ATCC stock strain was grown up overnight in 10 ml of DMB then serial diluted and plated on DMB agar. One unique colony was then selected and grown overnight in 10 ml of DMB to confluency and an aliquot was then used to perform a MIC assay to determine the sub-lethal concentration of FeSO_4_ to be utilized for selection purposes (3500 mg/l). A second aliquot was stored at –80°C in DMB supplemented with 50% glycerol and deemed the ancestral strain. The ancestral glycerol stock was then used to initiate a single 10 ml overnight culture in DMB, and the following day, 100 µl was used to inoculate each of ten 50 ml Erlenmeyer flask, 5 flasks with 9.9 ml of DMB to serve as the controls (C1–C5) and five flasks with 9.9 ml of DMB supplemented with 3500 mg/l FeSO_4_, to serve as the iron (II) selected populations (Fe^2+^_1–Fe^2+^_5). Each of the 10 populations were propagated daily for 200 days by subculturing 100 µl into 9.9 ml of their appropriate media. Every 7 days, glycerol stocks were made and frozen at –80°C for future genomic and phenotypic analysis. Of special note for this study, DMB medium contains the minimal amount of iron required for bacterial growth (0.1 µM or about 5.5 x 10^−3 ^mg/l) [28], thus the values listed for this metal are in addition to those present in the medium but in comparison to the concentration used for selection, is negligible.

### Twenty-four-hour growth assays

Twenty-four-hour growth assays were conducted in FeSO_4_ at multiple time points throughout the selection experiment to assess the acquisition of resistance. To perform the assay, the archived glycerol stocks were used to initiate 10 ml DMB ±3500 mg/l FeSO_4_ and grown overnight at 37°C. Overnight cultures were then diluted to an O.D._602 nm_ of 0.05 and added to each of the wells (in triplicate) of a 96-well plate containing a concentration gradient of FeSO_4_ (0–5000 mg/l) in DMB. The O.D._602 nm_ was taken at time = 0 h and time = 24-h and for analysis, the O.D._602 nm_ for 0–h data were subtracted from the 24-h data. Replicates were averaged and the means for each population were plotted in GraphPad Prism version 8.0.0 for Mac OS X (GraphPad Software, San Diego, CA, USA). Due to the nature of the serial dilution and the large number of data points under 100 mg/l, many graphs are represented on a log_10_ scale, for those graphs, 0 mg/ml was deemed as 1 in order for it to be represented on the plot. After 200-days, phenotypic assays were conducted to assess 24-h growth and potential pleiotropic effects associated with iron adaptation to both a variety of metals Fe^3+^, Ag^+^, Ga^3+^ and ${\text{SO}}_{4}^{–}$ and also to traditional antibiotics including ampicillin, sulfanilamide, polymyxin-B, rifampicin, bacitracin, chloramphenicol and tetracycline. Assays were performed as described above with test concentrations varying between 2500 and 0 mg/l depending on the toxicity of the compound. Statistical analysis of the effect of the selection regime [control or iron (II)-selected], concentration and their interaction for all 24-h growth data was performed via the General Linear Model utilizing SPSS version 23 (SPSS Inc, Armonk, NY, USA) and reported in Table 1. This is essentially a two-factor ANOVA with replication [29]. Phenotypic data will be submitted into DRYAD (https://datadryad.org/) upon acceptance of this manuscript for publication.

**Table 1.** *F* statistics and *P*-values for 24-h growth assays

Substance	Range tested	Population	Concentration	Interaction
Iron (II) resistant > controls				
Iron (II) FeSO_4_	6.2–2500 mg/l	*F *=* *179.1 *P *=* *0.0001	*F *=* *7.08 *P *=* *0.0001	*F *=* *1.26 *P *=* *0.265
Iron (III) Fe_2_(SO4)_3_	6.2–1750 mg/l	*F *=* *296.4 *P *=* *0.0001	*F *=* *10.5 *P *=* *0.0001	*F *=* *5.5 *P *=* *0.0001
Gallium Ga(NO_3_)_3._	6.2–5000 mg/l	*F *=* *886.4 *P *=* *0.0001	*F *=* *69.3 *P *=* *0.0001	*F *=* *2.5 *P *=* *0.006
Silver nitrate AgNO_3_	6.2–250 mg/l	*F *=* *1.059 *P *=* *0.307, NS	*F *=* *10.96 *P *=* *0.0001	*F *=* *0.359 *P *=* *0.951
Sodium sulfate NaSO^4^	6.2–5000 mg/l	*F *=* *133.5 *P *=* *0.0001	*F *=* *13.9 *P *=* *0.0001	*F *=* *6.2 *P *=* *0.0001
Ampicillin	6.2–2500 mg/l	*F *=* *10.1 *P *=* *0.0001	*F *=* *27.9 *P *=* *0.0001	*F *=* *20.7 *P *=* *0.0001
Bacitracin	6.2–2500 mg/l	*F *=* *353.9 *P *=* *0.0001	*F *=* *3.8 *P *=* *0.0001	*F *=* *29.3 *P *=* *0.0001
Chloramphenicol	6.2–2500 mg/l	*F *=* *147.3 *P *=* *0.0001	*F *=* *96.6 *P *=* *0.0001	*F *=* *39.2 *P *=* *0.0001
Polymixin-B	6.2–2500 mg/l	*F *=* *10.2 *P *=* *0.0001	*F *=* *113.9 *P *=* *0.0001	*F *=* *3.6 *P *=* *0.0001
Rifampicin	6.2–2500 mg/l	*F *=* *205.1 *P *=* *0.0001	*F *=* *23.2 *P *=* *0.0001	*F *=* *7.6 *P *=* *0.0001
Sulfanilamide	6.2–2500 mg/l	*F *=* *22.5 *P *=* *0.0001	*F *=* *95.5 *P *=* *0.0001	*F *=* *4.9 *P *=* *0.0001
Iron (II) resistant = controls				
Tetracycline	6.2–2500 mg/l	*F *=* *0.306 *P *=* *0.581	*F *=* *158.3 *P *=* *0.0001	*F *=* *0.245 *P *=* *0.951
Iron (II) resistant > ancestor				
Iron (II) FeSO_4_	6.2–5000 mg/l	*F *=* *154.4 *P *=* *0.0001	*F *=* *3.4 *P *=* *0.001	*F *=* *4.8 *P *=* *0.0001
Iron (III) Fe_2_(SO4)_3_	6.2–5000 mg/l	*F *=* *169.8 *P *=* *0.0001	*F *=* *3.5 *P *=* *0.001	*F *=* *18.07 *P *=* *0.0001
Gallium Ga(NO_3_)_3._	6.2–5000 mg/l	*F *=* *1107.3 *P *=* *0.0001	*F *=* *13.0 *P *=* *0.001	*F *=* *17.8 *P *=* *0.0001
Silver nitrate AgNO_3_	6.2–5000 mg/l	*F *=* *1.06 *P *=* *0.310, NS	*F *=* *5.58 *P *=* *0.001	*F *=* *2.04 *P *=* *0.109
Sodium sulfate NaSO^4^	6.2–5000 mg/l	*F *=* *136.6 *P *=* *0.0001	*F *=* *14.1 *P *=* *0.0001	*F *=* *6.5 *P *=* *0.0001
Ampicillin	6.2–2500 mg/l	*F *=* *206.8 *P *=* *0.0001	*F* = 60.4 *P *=* *0.0001	*F *=* *18.2 *P *=* *0.0001
Bacitracin	6.2–2500 mg/l	*F *=* *900.6 *P *=* *0.0001	*F *=* *28.3 *P *=* *0.0001	*F *=* *5.6 *P *=* *0.0001
Chloramphenicol	6.2–2500 mg/l	*F *=* *121.5 *P *=* *0.0001	*F *=* *94.7 *P *=* *0.0001	*F *=* *30.0 *P *=* *0.001
Polymixin-B	6.2–2500 mg/l	*F *=* *53.0 *P *=* *0.0001	*F *=* *112.7 *P *=* *0.0001	*F *=* *80.1 *P *=* *0.0001
Rifampicin	6.2–2500 mg/l	*F *=* *288.4 *P *=* *0.001	*F *=* *22.1 *P *=* *0.001	*F *=* *7.2 *P *=* *0.0001
Sulfanilamide	6.2–2500 mg/l	*F *=* *198.1 *P *=* *0.0001	*F *=* *98.4 *P *=* *0.0001	*F *=* *24.9 *P *=* *0.001
Tetracycline	6.2–2500 mg/l	*F *=* *35.4 *P *=* *0.0001	*F *=* *110.2 *P *=* *0.001	*F *=* *29.3 *P *=* *0.0001
Controls > ancestor				
Iron (II) FeSO_4_	6.2–1750 mg/l	*F *=* *5.2 *P *=* *0.02	*F *=* *2.3 *P *=* *0.01	*F *=* *2.5 *P *=* *0.01
Iron (III) Fe_2_(SO4)_3_	6.2–1750 mg/l	*F *=* *6.3 *P *=* *0.01	*F *=* *10.0 *P *=* *0.0001	*F *=* *8.6 *P *=* *0.0001
Gallium Ga(NO_3_)_3_	6.2–1000 mg/l	*F *=* *363.8 *P *=* *0.0001	*F *=* *76.0 *P *=* *0.0001	*F *=* *100.0 *P *=* *0.0001
Ampicillin	6.2–100 mg/l	*F *=* *69.7 *P *=* *0.0001	*F* = 13.8 *P *=* *0.0001	*F *=* *5.9 *P *=* *0.0001
Bacitracin	6.2–1000 mg/l	*F *=* *312.0 *P *=* *0.0001	*F *=* *3.3 *P *=* *0.001	*F *=* *30.0 *P *=* *0.0001
Polymixin-B	6.2–1000 mg/l	*F *=* *20.3 *P *=* *0.0001	*F *=* *119.6 *P *=* *0.0001	*F *=* *2.1 *P *=* *0.02
Rifampicin	6.2–1000 mg/l	*F *=* *95.8 *P *=* *0.001	*F *=* *48.3 *P *=* *0.001	*F *=* *13.2 *P *=* *0.0001
Sulfanilamide	6.2–1000 mg/l	*F *=* *95.8 *P *=* *0.0001	*F *=* *38.9 *P *=* *0.0001	*F *=* *5.3 *P *=* *0.0001
Tetracycline	6.2–1000 mg/l	*F *=* *53.0 *P *=* *0.0001	*F *=* *188.0 *P *=* *0.001	*F *=* *46.7 *P *=* *0.0001
Controls = ancestor				
Silver nitrate AgNO_3_	6.2–5000 mg/l	*F *=* *3.4 *P *=* *0.06	*F *=* *93.7 *P *=* *0.0001	*F *=* *17.8 *P *=* *0.0001
Sodium sulfate NaSO_4_	6.2–5000 mg/l	*F *=* *0.0 *P *=* *1.00	*F *=* *3.0 *P *=* *0.001	*F *=* *0.0 *P *=* *1.0000
Chloramphenicol	6.2–100 mg/l	*F *=* *0.59 *P *=* *0.44	*F *=* *48.1 *P *=* *0.0001	*F *=* *2.4 *P *=* *0.01

Notes: For statistical analysis, ANOVA using a generalized linear model was used to evaluate the treatment effect associated with iron (II) selection of the 24-h growth assays performed on the ancestral, control and adapted populations, (NS = not significant). The *F*-ratio is computed from the selection treatment MS/error MS; concentration MS/error MS and the interaction (selection x concentration) MS/error MS [29].

### Genomic analysis

DNA was extracted from all 10 populations at 200-days using the EZNA Bacterial DNA extraction kit (Omega Bio-tek^®^) as per manufacturer instructions. DNA concentrations were quantified using the QuantiFluor^®^ dsDNA system and genomic libraries were prepared using the Illumina Nextera XT kit. Libraries were sequenced on the Illumina MiSeq sequencing platform with depth of coverage ranging from ∼20x to ∼70x with all values reported in Supplementary Table S3. The sequences were deposited into the Sequence Read Archive (SRA) database under the accession code: PRJNA532971. Sequence alignment and variant calling from the samples was achieved by use of the *breseq* 0.35.2rc1 pipeline set to polymorphism mode (-p) and default parameters [30]. The *breseq* pipeline uses three types of evidence to predict mutations, read alignments, missing coverage and new junctions [28] and any reads that indicate a difference between the sample and the reference genome that cannot be resolved to describe precise genetic changes are listed as ‘unassigned’. These unassigned reads are not described nor interpreted here.

The initial *breseq* analysis showed that there was contamination in two of our iron selected populations (Fe^2+^_2 and Fe^2+^_5) by an unknown bacterium that was identified using the MetaPhlAn (version 2.0) pipeline to be the gram-negative bacterium, *Acinetobacter pittii*. The *breseq* pipeline was run a second time using both the *E. coli* K-12 MG1655 (NC_000913) and *A. pittii* ANC 4052 (NZ_KB976991) reference genomes. Of the 808 728 reads obtained for Fe^2+^_2, a total of 77% of the reads were able to be mapped, 70% of the mapped reads aligned to *E. coli’s* genome which has a genome size of ∼4.6 Mbp, in comparison to 30% of the reads aligned to *A. pittii* which has a genome size of ∼3.5 Mbp. In the case of Fe^2+^_5, there were a total of 1 006 649 reads of which a total of 75% could be mapped. Of the mapped reads, 73% mapped to the *E. coli* genome whereas 27% mapped to the *A. pittii* genome. Our confidence that adding the *A. pittii* ANC 4052 reference genome was effective in screening out incorrect variant calls is based on the similarity of our control results to our previous sequencing [31, 32] and the resulting iron selected variants identified with the alignments specific to *E. coli* occurred in genes known to be associated with iron and general metal metabolism. In addition, it is also worth noting that neither contaminated population showed any unique variation in phenotypic analysis.

## GENE EXPRESSION ANALYSIS

### Bacterial growth

After 200 days of selection, all 10 experimental (C1–C5 and Fe^2+^_1-Fe^2+^_5) populations were used for gene expression analysis. We did not evaluate the gene expression of the ancestral population as it showed neither adaptation to iron nor to the DMB medium. Thus, the comparison of gene expression between the Fe^2+^-selected and control populations allows for an evaluation of differences in gene expression patterns resulting from selection for FeSO_4_ resistance. Populations were grown overnight in standard DMB media, after overnight growth, they were diluted 1/100 into either DMB media alone or in DMB supplemented with 3500 mg/l of FeSO_4_. Cells were then grown at 37°C with shaking at 250 rpm. A total of 1 x 10^6^ cells were then harvested in mid-log phase at an O.D._600_ of 0.50. RNA was immediately extracted using the RNeasy^®^ Mini Kit (Qiagen) and quantified using the QuantiFluor^®^ RNA system and the Quantus^TM^ Fluorometer. RNA was then stored at –80°C prior to shipping to NanoString.

### nanoString gene expression analysis on the nCounter system

The *nCounter* assay uses a 35- to 50-base, color-coded probe pair to directly detect mRNA molecules of interest. The probe pair consists of a reporter probe which carries a unique color code at the 5’ end that enables molecular barcoding, and a capture probes which is linked to biotin at the 3’end for attachment of target genes during digital detection. To begin, the target mRNA is hybridized to the reporter-capture probes, excess probes are removed and the probe/target complexes are aligned and immobilized in the *nCounter* cartridge. This is then placed in a digital analyzer for image acquisition and data processing. Expression levels for a gene are measured by counting the number of times the color-coded barcode for that gene is detected [33]. Here, for the *nCounter* assay, samples were shipped to NanoString (nanostring.com, Seattle, WA, USA) and 100 ng of total RNA was then used for hybridization with the 50-gene custom designed code set and then processed according to manufacturer’s instruction. Raw RNA count data were normalized to average expression levels of two housekeeping genes (*hcaT* and *idnT*) using the nSolver^®^ analysis software. Normalized NanoString gene expression data were analyzed in terms of ratios and then converted into log_2_ ratios, (fold-changes). For statistical analysis, a *t*-test was performed on log_2_-transformed count data for each pairwise comparison and heat maps were then generated using GraphPad Prism 8 software to depict statistically significant fold-changes across these pairwise comparisons which were then further grouped by cellular function. The 48 genes were selected either because they acquired genetic mutations during the selection process or due to their involvement in one of five categories of biological processes that have been previously shown in the literature to be influenced by iron concentration. These five categories include: oxidative stress, biofilm formation, transport, metabolism and gene expression.

## RESULTS

### *Escherichia coli* can adapt to stressful levels of iron (II) sulfate [FeSO_4_]

**Figure 1.**
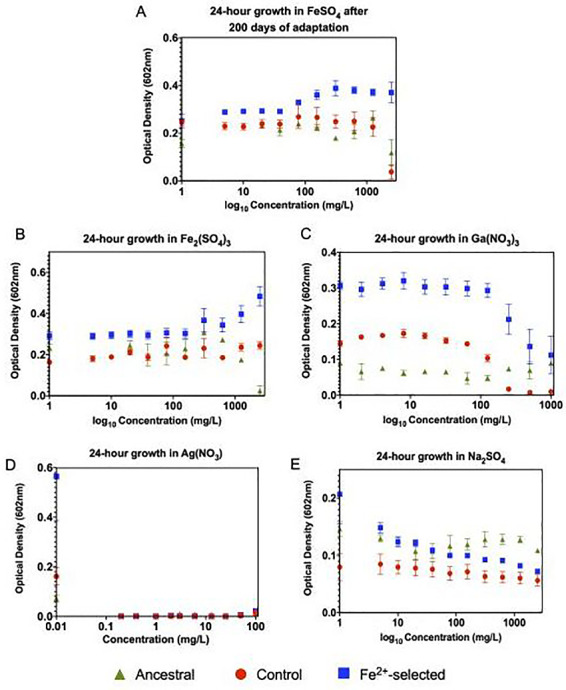
Twenty-four hours growth assay to assess the progression and overall timeframe of iron adaptation. All five individual control populations (C1–C5), all five individual iron adapted populations (Fe^2+^_1-Fe^2+^_5) and four replicates of the ancestral population were grown for 24-h in increasing concentrations of FeSO_4_ in triplicate. Optical densities were then measured at 602 nm. The mean of the controls (red), ancestral (green) and adapted (blue) populations were then plotted, error bars represent standard error among the populations. (**A**) After 200-days of adaptation, the control populations showed almost complete inhibition at 2500 mg/l. The iron (II)-selected populations display increasing optical densities at low concentration and steady growth at higher concentrations, all of which are significantly higher than what was exhibited by the controls and the ancestral population. This data giving strong evidence for the successful adaptation to optimized growth in iron (II) after 200-day of selection. (**B**) 24-h growth in Fe_2_(SO_4_)_3_, (**C**) 24-h growth in Ga(NO_3_)_3_, (**D**) 24-h growth in AgNO_3_ and (**E**) 24-h growth in Na_2_SO_4_. All iron (II)-adapted populations showed increased resistance to all four of the metals tested. Albeit, the overall growth rate increase was most apparent in iron (III) and the iron-analog gallium (III). Optical densities were severely hindered in both silver and sulfate with the iron (II)-adapted populations slightly outperforming the controls. This data demonstrates the pleiotropic effects associated with iron (II) adaptation which leads to general metal resistance. For statistical analysis, ANOVA analysis using a generalized linear model was performed to evaluate the treatment effect associated with selection in FeSO_4_. Both the F-statistic and *P*-values are reported in Table 1

After 200 days of selection, the iron (II)-selected populations displayed greater growth across all concentrations of FeSO_4_ compared to the controls and ancestor (Fig. 1A). This difference was extremely pronounced with increasing optical densities found at lower concentration (0–78 mg/l), and at higher concentrations showed steady growth attributable to a more constant outcome of optical densities. At 2500 mg/l the greatest difference was observed for iron (II)-selected > control > ancestor giving strong evidence for increased iron (II)-adaptation after 200 days of selection. The control (C1–C5) populations also showed significantly greater optical densities compared to the ancestral population with almost complete inhibition at 2500 mg/l. The *F* statistics and *P*-values are given for all phenotypic comparisons in Table 1a and b.

### Phenotypic results

The iron (II)-selected populations, along with the ancestral population were assessed for general metal resistance to determine potential pleiotropic effects associated with iron (II) adaptation (Fig. 1B–E). The iron (II)-selected populations showed greater growth compared to controls and the ancestor (Fe^2+^ > controls > ancestor) in two of the three metals tested (Fe^3+^ and Ga^3+^). All populations showed a reduction in growth with increasing concentration of metal and therefore demonstrated a significant concentration interaction for iron (III), gallium and silver (Table 1). Specifically, growth in iron (III) [Fe_2_(SO_4_)_3_] for all populations show steady optical densities across all concentrations (0–2500 mg/l), with the iron (II)-selected populations displaying a 1.5-fold increase in the median optical densities over the controls at low concentrations (0–125 mg/l) and a 2-fold increase at high concentrations (250–1000 mg/l) (Fig. 1B) with the ancestral strain completely inhibited at 2500 mg/l. Growth was then assessed in gallium (III) [Ga(NO_3_)_3_] across a narrower range of concentration (0–1000 mg/ml) due to its higher toxicity. The controls showed complete inhibition at 250 mg/l (Fig. 1C). In comparison, the iron (II)-selected populations which showed no significant reduction in optical densities until a concentration of 500 mg/l. The ancestral strain showed overall diminished growth compared to both iron (II)-selected and control populations. Next, growth was assessed in AgNO_3_ which showed no significant difference between any of the populations across all concentrations tested (0–1000 mg/l) (Fig. 1D). Finally, as a control, we assessed growth in sodium sulfate [Na_2_SO_4_] to assure that that effects observed in the iron (II)-selected populations were due to excess ionic iron and not the excess ionic sulfate (Fig. 1E). Here, we did observe a statistically significant change in growth iron (II) > controls > ancestral populations in the same concentration range used to assess FeSO_4_ (0–2500 mg/l). The difference between the iron (II)-selected populations and the controls was greater than the difference between controls and the ancestor, indicating iron (II) and iron (III) resistance are partially due to adaptation to sulfate.

### Iron (II) selection co-selects for general antibiotic resistance

**Figure 2.**
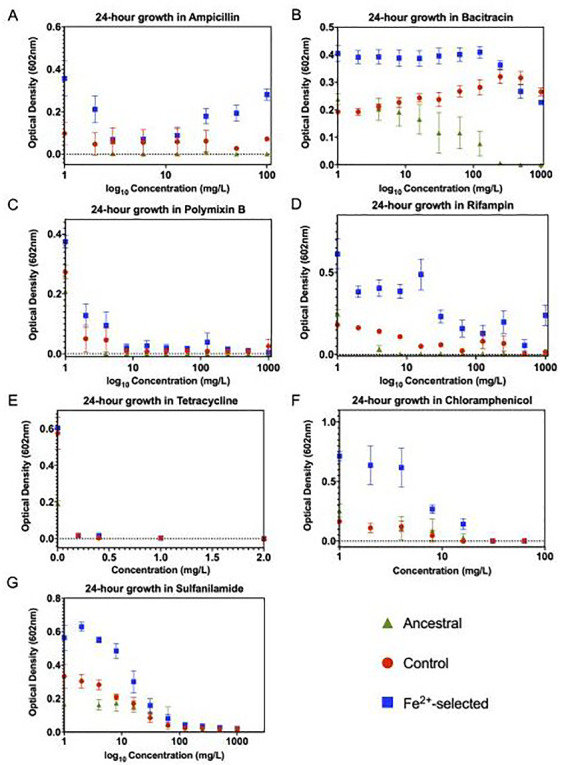
Twenty-four hours growth assays to assess general metal and antibiotic resistance associated with FeSO_4_ adaptation. Twenty-four hours optical assays were constructed similarly to those in Fig. 1 in seven antibiotics with varying mechanisms of action. (**A**) Ampicillin, (**B**) bacitracin, (**C**) polymyxin-B, (**D**) rifampin, (**E**) tetracycline (**F**) chloramphenicol and (**G**) sulfanilamide. All iron (II)-adapted populations showed increased resistance to four of the seven antibiotics tested. This data demonstrates the pleiotropic effects associated with iron (II) adaptation which leads to general antibiotic resistance. Statistical analysis was performed identically to Fig. 1 with both the F-statistic and *P*-values are displayed Table 1

As metal resistance has been shown to co-select for antibiotic resistance, we assessed growth in seven traditional antibiotics (Fig. 2). Twenty-four hours growth assays showed that the iron (II)-selected populations display greater growth compared to controls and the ancestor (Fe^2+^ > controls > ancestor) in all antibiotics tested except for tetracycline. Generally, populations showed a significant reduction of growth with increasing concentration of antibiotics, in addition to a significant population by concentration interaction (Table 1). This pattern was observed for the cell wall targeting antibiotics such as ampicillin, bacitracin and the cell membrane targeting antibiotic polymyxin-B (Fig. 2A–C), in addition to those antibiotics with an intracellular mode of action: chloramphenicol, rifampicin and sulfanilamide (Fig. 2D–F). Despite the statistical difference in optical densities evident at low concentrations of bacitracin, at the highest concentrations, it appears as though the controls have also evolved resistance as compared to the ancestral strains. This could indicate that resistance may be the result of alternative components of the media in the selection regime. In addition, we cannot conclude sulfanilamide resistance it is due specifically to iron selection as it could also be due to ${\text{SO}}_{4}^{2–}$ adaptation. Finally, tetracycline showed effective reduction in growth (O.D._602_ < 0.06) for all populations across all concentrations (0–100 mg/l) with no significant difference between the iron (II)-selected, controls and the ancestral populations (Fig. 2E).

### Genomic results

Following 200 days of selection, all populations were subjected to whole genome resequencing and compared to the *E. coli* K12 MG1655 sequence in the NCBI database (NC_000913) to detect any polymorphisms [indels and single nucleotide polymorphisms (SNPs)] associated with their selection regime. Ancestral differences have already been reported in our previous work [31, 32], and those ancestral mutations detected in populations sequenced here are reported in Supplementary Table S1.

Selective sweeps in the iron (II)-selected populations were observed at day 200 with all frequencies of mutation (*f*) reported in Table 2 and positional coverage of each mutation is reported in Supplementary Table S3. Specifically, these sweeps occurred in: *ptsP*, *ilvG* and *fecA*. Three different mutations were detected in the *ptsP* gene, both Fe^2+^_1 and Fe^2+^_2 carried a premature stop codon at position 519, with a *f *=* *1.00, whereas Fe^2+^_3 carried three different *ptsP* mutations within the population, R256C, C519* and a 1 bp deletion, with *f = *0.155, 0.491 and 0.226, respectively. Three of the five populations (Fe^2+^_1–3) carried hard sweeps in *ilvG,* all of them being a 1 bp insertion. One of the populations that did not acquire an *ilvG* mutation did have an intergenic mutation between *ilvL* and *ilvX* to a *f = *1.00. Finally, three different *fecA* SNPs were detected in four of the five populations. A559T was found in both Fe^2+^_1 and Fe^2+^_2, both at a *f *=* *1.00, whereas Fe^2+^_3 acquired a D120Y mutation albeit at a lower frequency (*f* =* *0.327). Finally, Fe^2+^_5 carried the G243C mutation (*f *=* *1.000), and after 200-days of selection Fe^2+^_4 is the only population to not harbor a *fecA* mutation. Three of our lines did carry identical mutations, most notably in Fe^2+^_1 and Fe^2+^_2 which all had a *f = *1.00 in *ptsP, fecA* and *ilvG*. We acknowledge that this could have occurred due to contamination between the populations, but we believe that it is more likely nucleotide parallelism. Our assumptions are supported by our previous work [31, 32] in which we observed single-nucleotide parallelism as a result of silver adaptation among many of our populations.

**Table 2.** Genomic adaptation in FeSO_4_ selected populations

Gene	Mutation	Fe2+_1	Fe2+_2	Fe2+_3	Fe2+_4	Fe2+_5	Gene description *BreSeq and Uniprot
***murC*→**	**P14S (CCC→TCC)**	**0.208**	**––**	**––**	**––**	**––**	Cell wall formation, UDP-N-acetylmuramate: L-alanine ligase
***cueR*→**	**V6L (GTA→CTA)**	**––**	**––**	**––**	**––**	**0.747**	Copper-responsive regulon transcriptional regulator
***mrdA*←**	**G69R (GGC→CGC)**	**––**	**––**	**0.348**	**––**	**––**	Transpeptidase involved in peptidoglycan synthesis (penicillin-binding protein 2)
***mdfA*→**	**E250G (GAG→GGG)**	**––**	**––**	**––**	**0.197**	**––**	Multi-drug efflux system protein
***yeaG*→**	**A441V (GCA→GTA)**	**––**	**––**	**––**	**––**	**1.000**	Protein kinase, endogenous substrate unidentified; autokinase
***ptsP*←**	**R526C (CGC→TGC)**	**––**	**––**	**0.155**	**––**	**––**	Component of the PEP-dependent nitrogen-metabolic phosphotransferase system
***ptsP*←**	C519*(TGC→TGA)	**1.000**	**1.000**	**0.491**	**––**	**––**	Component of the PEP-dependent nitrogen-metabolic phosphotransferase system
***ptsP*←**	**(Δ1 bp) coding (1525/2247 nt)**	**––**	**––**	**0.226**	**––**	**––**	Component of the PEP-dependent nitrogen-metabolic phosphotransferase system
***yhfZ*←/ ←*trpS***	**intergenic (–223/+67)**	**––**	**––**	**0.121**	**––**	**––**	Putative DNA-binding transcriptional regulator/tryptophanyl-tRNA synthetase
***yhfZ*←/ ←*trpS***	**intergenic (–242/+48)**	**––**	**––**	**0.135**	**––**	**––**	Putative DNA-binding transcriptional regulator/tryptophanyl-tRNA synthetase
***rhsB*→**	**K1374N (AAG→AAT)**	**––**	**––**	**0.229**	**––**	**––**	Rhs family putative polymorphic toxin, putative neighboring cell growth inhibitor
***yidX*→**	**L29V (CTG→GTG)**	**––**	**1.000**	**––**	**––**	**––**	Putative lipoprotein
***yidX*→**	**coding (87–88/657 nt)**	**––**	**1.000**	**––**	**––**	**––**	Putative lipoprotein
***rrsC*→**	**noncoding (226/1542 nt)**	**––**	**––**	**––**	**––**	**1.000**	16S ribosomal RNA of rrnC operon
***ilvL*→/ →*ilvX***	**intergenic (+46/–41)**	**––**	**––**	**––**	**––**	**1.000**	Biosynthesis of branched-chain amino acids,
***ilvG*→**	**(+C) pseudogene (66/663 nt)**	**1.000**	**1.000**	**0.667**	**––**	**––**	Branched-chain amino acids synthesis
***rpoB*→**	**D654Y (GAC→TAC)**	**––**	**––**	**––**	**0.377**	**––**	RNA polymerase, beta subunit
***fecA*←**	**A559T (GCT→ACT)**	**1.000**	**1.000**	**––**	**––**	**––**	Fe3 + dicitrate siderophore transport system
***fecA*←**	**G243C (GGC→TGC)**	**––**	**––**	**––**	**––**	**1.000**	Fe3 + dicitrate siderophore transport system
***fecA*←**	**D120Y (GAC→TAC)**	**––**	**––**	**0.327**	**––**	**––**	Fe3 + dicitrate siderophore transport system

Notes: After 200 days of adaptation, all populations were subjected to whole genome resequencing with sequence alignments and variant calling using *breseq* 0.30.0. Mutations found in both the controls and iron (II)-adapted populations were removed (reported in Supplementary Table S1). The genes/mutations highlighted in orange represent those that were identified in more than one population. In addition to the gene name, the corresponding protein and nucleotide changes are also reported along with the frequency of mutation (f), represented as a value from 0 (not detected, gray box)––1.000 (hard sweep and dark green) with minor variants colored in lighter shades of green. NCBI SRA database PRJNA532971.

Additionally, three genes: *yeaG*, *yidX* and *rrsC* showed hard sweeps in one of the five iron (II)-selected populations along with a number of mutations detected at lower frequencies. Other than the reported *ptsP* mutation, which was maintained in the selected populations and lost in the controls by day 200, none of the suspected mutations associated with iron (II)-selection were found in the controls, nor have ever been observed in the control or ancestral populations of our previous studies [31, 32]. All polymorphisms called by *breseq* 3.0.0 in the controls and the iron (II)-selected populations are found in Supplementary Table S1.

An earlier sequencing run of our lineages (day-51) detected the presence of a contaminant in a few of our lines, and by day-200, as described, detection of that contaminant remained present in two of our iron selected populations (Fe^2+^_2 and Fe^2+^_5). As a result, the *breseq* pipeline was run using both the *E. coli* K-12 MG1655 (NC_000913) and *A. pittii* ANC 4052 (NZ_KB976991) reference genomes to screen out incorrect variant calls. We acknowledge that it is possible that this contaminant may have influenced the evolutionary trajectory of our populations by selecting for other mutations that may produce a fitness advantage in presence of iron. Based on the similarity of our control results to our previous sequencing [31, 32] and the resulting iron selected variants identified with the alignments specific to *E. coli* occurring in genes known to be associated with iron and general metal metabolism we believe that our mutations are likely due to iron adaptation.

### Gene expression

**Figure 3.**
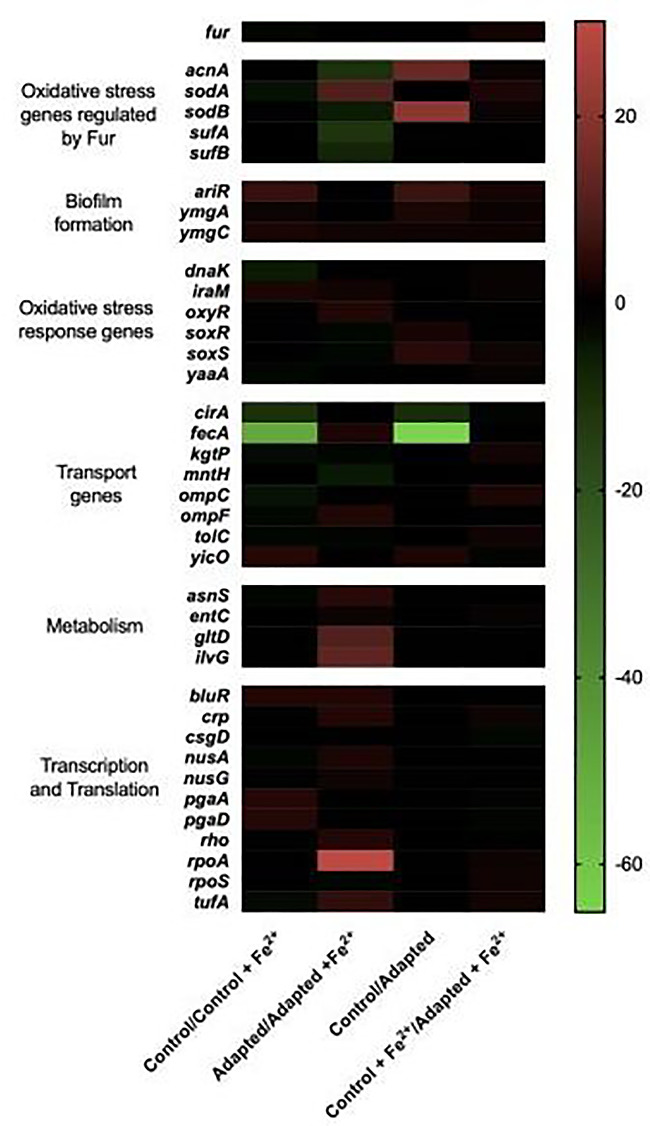
Differential gene expression in response to FeSO_4_ acclimation. Gene expression studies were conducted at day 200 using NanoString technologies of 48 genes selected for their acquisition of mutations during the selection regime or for their role in general iron metabolism. Here, fold changes in RNA counts between each averaged set of populations for 38 of the 50 selected genes are reported (the remainder can be seen in Supplementary Table S2). For analysis, all genes that had a calculated *P*-value > 0.05 for their fold change were deemed zero. Each averaged population [controls or iron (II) adapted] were analyzed in pairwise combinations for fold-changes and represented in the heat map. Most notably, the adapted populations’ downregulate genes involved in iron transport and increase expression of oxidative stress response genes even in absence of exposure to iron. Normalized NanoString gene expression data was analyzed in terms of ratios and then converted into log_2_ ratios, (fold-changes). For statistical analysis, a *t*-test was performed on log_2_-transformed count data for each pairwise comparison and reported in Supplementary Table S2

After obtaining the sequencing results, we then assessed changes in gene expression that result from iron adaptation for a selected subset of genes. Four genes were selected as they acquired mutations during adaptation (*ptsP, ilvG, fecA* and *murC*). We then surveyed the literature for biological processes that are influenced by cellular iron. These biological processes were determined to be: oxidative stress, biofilm formation, transport, metabolism and gene expression. We then chose 44 genes that then fell across those categories, again based on the iron literature, in addition to two housekeeping genes: *hcaT* and *idnT*. These were then used to assess differential gene expression in the 200-day selected and control populations in an effort to better understand their differences in cellular response to iron (II). The controls (C1–C5) were chosen over the ancestral population for these studies as they have adapted to growth in the same DMB media for the same length of time as the iron (II)-selected populations, making FeSO_4_ the only difference between their potential cellular responses. For analysis, each of the 48 genes were normalized against 2 housekeeping genes (*hcaT* and *idnT*). All genes that showed no significant change (*P* > 0.05) as evaluated by nSolver were set to zero and only those showing a 2-fold or greater change (in at least one pair) are represented in Fig. 3 (for a complete list of results for all 50 genes and their corresponding *P*-values see Supplementary Table S2). Both housekeeping genes showed no significant change (*P* = 0.8) as expected.

During log phase growth, control populations show a few changes worth noting in response to the addition of FeSO_4_. First, they show a 2.2-fold reduction in *fur* expression and almost no changes in genes associated with oxidative stress other than a 3.9-fold reduction in *sodA*. The largest response is in the expression of iron transport genes, specifically, reducing the iron-siderophore transporters *fecA* and *cirA* expression by 50-fold and 11-fold, respectively, and outer membrane porins *ompC* and *ompF* by 3- and 4-fold, respectively. These populations also show a 2-fold reduction in *tolC* which aids in export of siderophores. Finally, these populations show an increase in all three-iron stress associated biofilm genes, *ariR*, *ymgA* and *ymgC* by 6-, 2- and 2.4-fold, respectively. When the FeSO_4_ response in the adapted population is compared to the FeSO_4_ response in the controls it is evident that there are few changes in the overall response (upon exposure) indicating that both populations have similar overall responses.

The most notable differences lie in comparisons between the control and iron (II)-selected populations in absence of FeSO_4_. Here, the adapted populations show four important clusters. First regarding oxidative stress, four genes, *acnA, sodB, soxS* and *soxR* all show a significant increase in expression, 14-, 20- 4- and 4-fold, respectively and, second, there is an overexpression of three genes involved in biofilm formation, *ariR* (6.8-fold), *ymgA* (2.6-fold) and *ymgC* (1.8-fold). Third, despite small changes in transport protein expression upon FeSO_4_ exposure, in its absence, the iron (II)-selected populations have already downregulated both *fecA* 65-fold and *cirA* 10-fold in comparison to the controls, indicating that selected populations have potentially used a pre-emptive cellular response prior to exposure. Finally, along with this, they also show evidence of an increased transcriptional and translational response upon exposure to FeSO_4_. This includes a 30-fold increase the *rpoA* expression, a 3.5-fold increase in *rpoS* and a 5-fold increase in *tufA.* None of these genes increase expression in the control populations, and this therefore shows further genetic evidence for iron adaptation that may correlate with the increased optical densities observed in the 24-h growth assays in FeSO_4_ over the control and the ancestral populations.

## CONCLUSIONS

Iron limitation remains one of the most important nutritional stresses limiting bacterial growth, and therefore mechanisms of acquisition under iron starvation remain an intense area of interest. Unfortunately, the response used by bacteria to acclimate and adapt under conditions of iron intoxication are less well known and understood. In our studies, natural selection in excess iron (II), favored clones with genomic variants allowing the capacity to modulate iron metabolism to acquire the required amount for growth, yet minimizing damage from intoxication. Conversely, natural selection in the control environment favored genomic variants that allow gene expression patterns tuned to the acquisition of scare iron; as DMB medium contains barely enough iron to allow bacterial growth [32]. This is evidenced by the fact that in both the iron (II, III) assays, growth of controls and iron (II)-selected populations increased at the lowest concentrations of additional iron. This being said, iron (II)-adapted populations also show superior growth over the controls in absence of FeSO_4_ and may be due to the increased transcription/translation response but still remains to be further characterized. As we do not know how long physiological acclimation takes relative to iron concentration. It is likely that the gene expression patterns we observed reflect the evolved pattern for each population in its culture environment. Further understanding of this survival dynamic could be attained by investigations regarding how long physiological acclimation takes relative to iron concentration, which would yield insight on the effects of phenotypic plasticity across the different stages of this evolutionary experiment.

**Figure 4.**
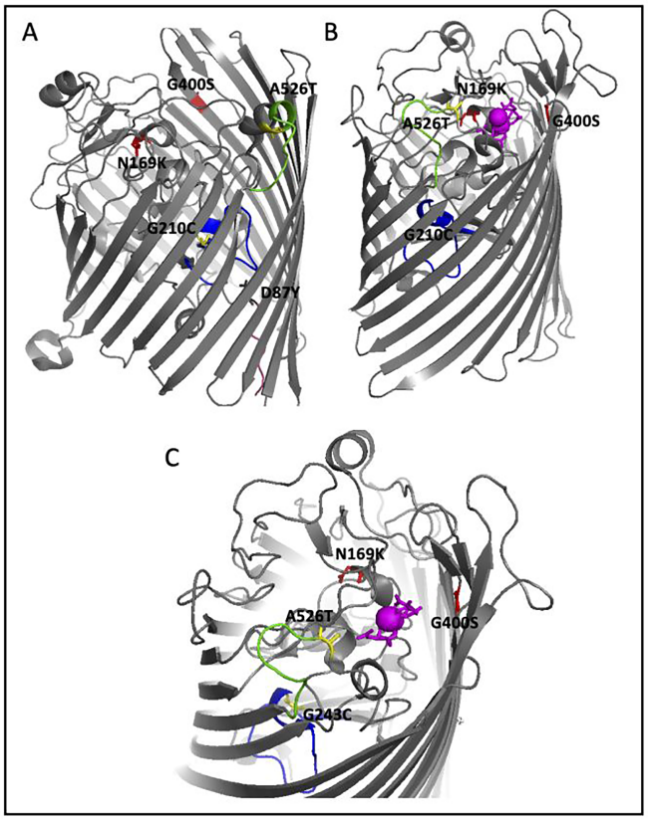
Mapping of the FecA mutations. Sequencing of the 200-day iron (II)-adapted populations identified three mutations in the *fecA* genes (D87Y, G201C and A526T). (**A**) The apo-FecA structure (PDB1PNZ) was used to map these three residues. D87Y (black) which is unique to the 200-day populations resides adjacent to the TonB-binding box (pink), G210C (yellow) in the internal plug domain important for ligand transport (blue) and A526T (yellow) on the periplasmic lid (green). Both G210C and A526T were also detected in our 45-day iron (II) adapted populations [27]. In red are two mutations (N169K and G400S) which were detected in gallium (iron analog) resistant strains [38] and as shown, map to similar regions as the iron resistant mutations. (**B**) The iron-citrate complexed structure (PDB1PO3) was also used to map two of the three iron resistant residues (D87Y lies in an unstructured region and therefore could not be mapped) and the gallium mutations. This shows the major rearrangement of the periplasmic lid (green) and the significant movement of A526 upon ligand binding. (**C**) The iron-citrate binding site of the bound structure (PDB1PO3) to demonstrate the proximity of two of the mapped residues (G210C and A526T) and the gallium mutations (N169K and G400S) to the ligand binding site. The importance of all three regions indicate that these mutations may prevent iron-citrate transport and therefore limit entry of excess iron into the cell by occlusion thereby increasing chances of survival and evidence for genetic adaptation

This work shows that we were able to show that *E. coli* K-12 MG1655 can acclimate to stressful levels of iron (II). Our data indicates that this may happen through decreasing expression of iron transport genes and increasing iron related biofilm genes, although prolonged exposure will likely lead to decreased viability and death. Alternatively, as we showed here, *E. coli* can rapidly adapt to survive under FeSO_4_ intoxication requiring only a small number of genomic changes, one most notably in *fecA*. FecA is a membrane bound iron-citrate transporter and its relationship to iron metabolism has been well established [34, 35]. We suggest here that *fecA* may be the main response by which the cell prevents entry of iron in our experimental environment, thereby decreasing its toxic effects and potential for Fenton chemistry. DMB contains 0.5 g/l of sodium citrate, the siderophore necessary for iron import by *fecA* further supporting this hypothesis. In this study, we showed three separate mutations in regions that have been shown to be essential for function [36, 37] (Fig. 4) and were present in four of the five populations. Selected populations also showed decreased expression in *fecA*.

One of the most impactful findings is that our selection regime also conferred resistance to other metals (gallium and ferric sulfate) and to as many as six traditional antibiotics with different modes of action (ampicillin, bacitracin, polymyxin-B, rifampin, sulfanilamide and chloramphenicol). The findings observed for the metals was not surprising—*E. coli* is known to have a similar cellular response to ferric iron as it does with ferrous iron thereby allowing resistance of one to confer resistance to both. Second, gallium is an iron analog. In our previous work, this strain of *E. coli* also acquires mutations in *fecA* as a result of gallium resistance which could account for the increased optical densities in the adapted strain in the metal [38]. What is most concerning is the multi-drug resistance acquired in this strain, which indicates that these strains have the potential to be even more harmful than originally anticipated.

Since, adjusting to environmental changes by altering gene expression can take time, it would be advantageous for survival to pre-emptively prepare for nutritional stresses when possible [1]. Specifically, in absence of FeSO_4_, the iron (II)-selected populations showed a differential cellular response over the controls by having constitutively increased levels of oxidative stress genes, decreased levels of transport genes and increased levels of iron-stress biofilm genes. This may indicate that the iron (II)-selected populations have evolved the ability to endure the effects of iron intoxication as part of their normal developmental process as they are actively replicating [39]. Future genomic and expression analysis through whole transcriptome RNAseq studies may provide additional insight into the evolution of this efficient mechanism of adaptation and provide a broader overview of the adapted cellular response.

## IMPLICATIONS

With the rise of antibiotic resistance and the search for alternative methods of control, these results should highlight the role of excess ionic and nanoparticle iron to target pathogenic and multi-drug resistant bacteria as the implementation of such approaches without reference to basic evolutionary principles make them doomed to fail.

## AUTHOR CONTRIBUTIONS

M.D.T. wrote the first draft of the manuscript and contributed to the interpretation of all data. M.D.T., A.J.E., D.K.W., A.M., S.B. and A.T. were responsible for conducting experiments. K.L.R., S.H., A.M. and J.L.G. performed all software analysis. M.D.T. oversaw the work of D.K.W. and A.M. J.L.G. oversaw the work of A.J.E., S.B. and A.T. J.L.G. acquired funding, contributed conception and design of the study in addition to the analysis and interpretation of all data, performed all of the statistical analysis. All authors contributed to the manuscript revision, read and approved the submitted version.

## Supplementary Material

eoaa051_Supplementary_Data
