# Supplementary material for: Too much of a good thing: Adaption to iron (II) intoxication in Escherichia coli
Source: Evol Med Public Health. 2021 Jan 18;9(1):53–67. doi: 10.1093/emph/eoaa051 (PMC7937436; doi:10.1093/emph/eoaa051)
Supplement: eoaa051_Supplementary_Data [file eoaa051_supplementary_data.zip › Supplemental_Table_3.docx]

**Supplemental Table 3** – **Sequencing coverage.** Here we report the total reads for each adapted lineage and indicate the % of the total mapped reads which correspond to both our target species, *Escherichia coli* and for the contaminant *Acinetobacter pittii*. In addition, we report the positional coverage for each of the high frequency mutation to validate our conclusions derived from these results. * the positional coverage is not reported by breseq for samples containing more than one species.

| **Total sequencing coverage for the population** | | **Fe2+_1** | **Fe2+_2** | **Fe2+_3** | **Fe2+_4** | **Fe2+_5** |
| --- | --- | --- | --- | --- | --- | --- |
| ***Total reads*** | | **1,354,411** | **808,728** | **676,049** | **671,878** | **1,006,649** |
| ***total % of reads mapped*** | | 91.4 | 77.2 | 82.6 | 86.3 | 75.1 |
| ***% reads mapped to Escherichia coli*** | | 100 | 70 | 100 | 100 | 72.6 |
| ***% reads mapped to Acinetobacter pittii*** | | 0 | 30 | 0 | 0 | 24.7 |
| **Gene** | **Mutation** | **Positional Coverage for each high frequency mutation** | | | | |
| ***murC* →** | **P14S (CCC→TCC)** | **77x** |  |  |  |  |
| ***cueR* →** | **V6L (GTA→CTA)** |  |  |  |  | ***** |
| ***mrdA* ←** | **G69R (GGC→CGC)** |  |  | **37x** |  |  |
| ***mdfA* →** | **E250G (GAG→GGG)** |  |  |  | **29x** |  |
| ***yeaG* →** | **A441V (GCA→GTA)** |  |  |  |  | ***** |
| ***ptsP* ←** | **R526C (CGC→TGC)** |  |  | **33x** |  |  |
| ***ptsP* ←** | **C519* (TGC→TGA)** | **61x** | ***** | **33x** |  |  |
| ***ptsP* ←** | **(Δ1 bp) coding (1525/2247 nt)** |  |  | **31x** |  |  |
| ***yhfZ* ← / ← *trpS*** | **intergenic (‑223/+67)** |  |  | **33x** |  |  |
| ***yhfZ* ← / ← *trpS*** | **intergenic (‑242/+48)** |  |  | **36x** |  |  |
| ***rhsB* →** | **K1374N (AAG→AAT)** |  |  | **55x** |  |  |
| ***yidX* →** | **L29V (CTG→GTG)** |  | ***** |  |  |  |
| ***yidX* →** | **coding (87‑88/657 nt)** |  | ***** |  |  |  |
| ***rrsC* →** | **noncoding (226/1542 nt)** |  |  |  |  | ***** |
| ***ilvL* → / → *ilvX*** | **intergenic (+46/‑41)** |  |  |  |  | ***** |
| ***ilvG* →** | **(+C) pseudogene (66/663 nt)** | **62x** | ***** | **27x** |  |  |
| ***rpoB* →** | **D654Y (GAC→TAC)** |  |  |  | **32x** |  |
| ***fecA* ←** | **A559T (GCT→ACT)** | **65x** | ***** |  |  |  |
| ***fecA* ←** | **G243C (GGC→TGC)** |  |  |  |  | ***** |
| ***fecA* ←** | **D120Y (GAC→TAC)** |  |  | **34x** |  |  |
