# Supplementary material for: Too much of a good thing: Adaption to iron (II) intoxication in Escherichia coli
Source: Evol Med Public Health. 2021 Jan 18;9(1):53–67. doi: 10.1093/emph/eoaa051 (PMC7937436; doi:10.1093/emph/eoaa051)
Supplement: eoaa051_Supplementary_Data [file eoaa051_supplementary_data.zip › Supplemental_Table 1.docx]

**Supplemental Table 1 -** After 200-days of adaptation, the populations (iron (II)-adapted and controls) were subjected to whole genome resequencing using the Illumina MiSeq sequencing platform with sequence alignments and variant calling achieved using the *breseq* 0.30.0 pipeline. All of the detected polymorphisms have been reported in the tables below, the ancestral strain was sequenced in our previous work. The gene names, the corresponding protein and nucleotide changes are reported along with the frequency of mutation (*f*), for intergenic mutations, both the chromosomal position and observed mutation are also reported under annotation. In addition, it is important to note that there are some changes between our ancestral strains (ATCC #47076) that was used to begin the evolution experiments and the reference genome (NC_000913), these are reported under “Ancestral”. These mutations were first identified in our previous work where we sequenced the ancestral strain [31] and therefore those particular mutations are separated from the general sequence results, it is also important to note that there are slight differences between each data set as the depth of coverage varied between samples.

| **Polymorphisms in the iron (II)-selected populations at day 200.** | | | | | | | |
| --- | --- | --- | --- | --- | --- | --- | --- |
| **Gene** | **Annotation** | **Fe^2+^_1** | **Fe^2+^_2** | **Fe^2+^_3** | **Fe^2+^_4** | **Fe^2+^_5** | **Description** |
| *caiD* ← / ← *caiC* | intergenic (‑26/+83), 36,188, A→T | 0.067 | 0.000 | 0.000 | 0.000 | 0.000 | carnitinyl‑CoA dehydratase/putative crotonobetaine /carnitine‑CoA ligase. |
| *kefC* → | S172G (AGC→GGC) | 0.000 | 0.207 | 0.000 | 0.000 | 0.000 | potassium: proton antiporter |
| *murC* → | P14S (CCC→TCC) | 0.208 | 0.000 | 0.000 | 0.000 | 0.000 | UDP‑N‑acetylmuramate:L‑alanine ligase |
| *rrsH* → | noncoding  (1120/1542 nt)  224,890, T→C | 0.000 | 0.000 | 0.168 | 0.000 | 0.000 | 16S ribosomal RNA of *rrnH* operon |
| *rrsH* → | 224,907, C→A | 0.000 | 0.000 | 0.128 | 0.000 | 0.000 | 16S ribosomal RNA of *rrnH* operon |
| *rrsH* → | 224,910, C→G | 0.000 | 0.000 | 0.124 | 0.000 | 0.000 | 16S ribosomal RNA of *rrnH* operon |
| *mmuM* → | E97G (GAG→GGG) | 0.121 | 0.000 | 0.000 | 0.000 | 0.000 | CP4‑6 prophage; S‑methylmethionine:homocysteine methyltransferase. |
| *yagH* → | S170P (TCG→CCG) | 0.108 | 0.000 | 0.000 | 0.000 | 0.000 | CP4‑6 prophage; putative xylosidase/arabinosidase |
| *yagJ* → | pseudogene (231/828 nt)291,634, G→T | 0.000 | 0.202 | 0.000 | 0.000 | 0.000 | CP4‑6 prophage; uncharacterized protein; Phage or Prophage related. |
| *citA* → | D475Y (GAT→TAT) | 0.060 | 0.000 | 0.000 | 0.000 | 0.000 | sensory histidine kinase in two‑component regulatory system with CitB |
| *mrdA* ← | G69R (GGC→CGC) | 0.000 | 0.000 | 0.348 | 0.000 | 0.000 | Transpeptidase involved in peptidoglycan synthesis (penicillin‑binding protein 2) |
| *kdpD* ← | V138G (GTT→GGT) | 0.000 | 0.000 | 0.084 | 0.000 | 0.000 | fused sensory histidine kinase in two‑component regulatory system with KdpE: signal sensing protein |
| *ybfD* → | I152I (ATC→ATT) | 0.000 | 0.000 | 0.000 | 0.198 | 0.000 | H repeat‑associated putative transposase |
| *phr* → | L356L (TTA→TTG) | 0.000 | 0.000 | 0.082 | 0.000 | 0.000 | deoxyribodipyrimidine photolyase, FAD‑binding |
| *ybhM* → | A105T (GCC→ACC) | 0.000 | 0.000 | 0.141 | 0.000 | 0.000 | UPF0005 family inner membrane protein |
| *mdfA* → | E250G (GAG→GGG) | 0.000 | 0.000 | 0.000 | 0.197 | 0.000 | multidrug efflux system protein |
| *ybjT* ← / ← *ltaE* | intergenic (‑4/+7), 908,286, T→C | 0.109 | 0.000 | 0.000 | 0.000 | 0.000 | putative NAD‑dependent oxidoreductase/L‑allo‑threonine aldolase, PLP‑dependent |
| *hyaB* → | G177G (GGG→GGT) | 0.000 | 0.000 | 0.000 | 0.134 | 0.000 | hydrogenase 1, large subunit |
| *ymdA* → /  → *ymdB* | intergenic (+7/‑88) 1,105,732, G→T | 0.000 | 0.188 | 0.000 | 0.000 | 0.000 | uncharacterized protein/O‑acetyl‑ADP‑ribose deacetylase; RNase III inhibitor during cold shock; putative cardiolipin synthase C regulatory subunit |
| *icd* → | D168Y (GAC→TAC) | 0.055 | 0.000 | 0.000 | 0.000 | 0.000 | e14 prophage; isocitrate dehydrogenase, specific for NADP+ |
| *lit* → | N195D (AAC→GAC) | 0.000 | 0.138 | 0.000 | 0.000 | 0.000 | e14 prophage; cell death peptidase, inhibitor of T4 late gene expression |
| *ymfI* → | D112Y (GAT→TAT) | 0.051 | 0.000 | 0.000 | 0.000 | 0.000 | e14 prophage; uncharacterized protein |
| *galU* → / ← *hns* | intergenic (+81/+63) 1,292,446, T→C | 0.000 | 0.000 | 0.000 | 0.091 | 0.000 | glucose‑1‑phosphate uridylyltransferase/global DNA‑binding transcriptional dual regulator H‑NS |
| *hrpA* → | A671V (GCG→GTG) | 0.067 | 0.000 | 0.000 | 0.000 | 0.000 | putative ATP‑dependent helicase |
| *ydcS* → | G320G (GGC→GGA) | 0.059 | 0.000 | 0.000 | 0.000 | 0.000 | polyhydroxybutyrate (PHB) synthase, ABC transporter periplasmic binding protein homolog |
| *ydcC* → | L277P (CTC→CCC) | 0.000 | 0.000 | 0.124 | 0.000 | 0.000 | H repeat‑associated putative transposase |
| *ydfJ* ← / → *ydfK* | intergenic (‑701/‑86) 1,632,986, G→A | 0.109 | 0.000 | 0.000 | 0.000 | 0.000 | pseudogene, MFS transporter family; interrupted by Qin prophage Phage or Prophage Related; putative transport protein/cold shock protein, function unknown, Qin prophage |
| *slyA* ← | G6G (GGT→GGA) | 0.053 | 0.000 | 0.000 | 0.000 | 0.000 | global transcriptional regulator |
| *ydiO* → | *384Y (TAA→TAT) | 0.094 | 0.000 | 0.000 | 0.000 | 0.000 | putative acyl‑CoA dehydrogenase |
| *yeaG* → | A441V (GCA→GTA) | 0.000 | 0.000 | 0.000 | 0.000 | 1.000 | protein kinase, endogenous substrate unidentified; autokinase |
| *proQ* ← | H177H (CAC→CAT) | 0.000 | 0.000 | 0.000 | 0.167 | 0.000 | RNA chaperone, putative ProP translation regulator |
| *torZ* ← | M374R (ATG→AGG) | 0.000 | 0.199 | 0.000 | 0.000 | 0.000 | trimethylamine N‑oxide reductase system III, catalytic subunit |
| *atoS* → | G134S (GGT→AGT) | 0.118 | 0.000 | 0.000 | 0.000 | 0.000 | sensory histidine kinase in two‑component regulatory system with AtoC |
| *nuoC* ← | S314I (AGT→ATT) | 0.000 | 0.000 | 0.000 | 0.146 | 0.000 | NADH:ubiquinone oxidoreductase, fused CD subunit |
| *yfeZ* ← | L21L (TTG→CTG) | 0.000 | 0.145 | 0.000 | 0.000 | 0.000 | inner membrane protein |
| *hcaT* ← | P291S (CCG→TCG) | 0.000 | 0.000 | 0.139 | 0.000 | 0.000 | putative 3‑phenylpropionic transporter |
| *yphA* → / ← *yphB* | intergenic (+16/+32), 2,673,784, C→T | 0.000 | 0.000 | 0.000 | 0.134 | 0.000 | DoxX family inner membrane protein/mutarotase superfamily protein, YphB family |
| *aroF* ← / → *yfiL* | intergenic (‑96/‑114) 2,741,246, G→T | 0.000 | 0.000 | 0.000 | 0.000 | 0.127 | 3‑deoxy‑D‑arabino‑heptulosonate‑7‑phosphate synthase, tyrosine‑repressible/lipoprotein |
| *ygcS* ← | M49T (ATG→ACG) | 0.064 | 0.000 | 0.000 | 0.000 | 0.000 | putative transporter |
| *ygdH* → | D123N (GAC→AAC) | 0.000 | 0.099 | 0.000 | 0.000 | 0.000 | UPF0717 family protein |
| *ptsP* ← | R526C (CGC→TGC) | 0.000 | 0.000 | 0.155 | 0.000 | 0.000 | fused PTS enzyme: PEP‑protein phosphotransferase (enzyme I)/GAF domain containing protein |
| *ptsP* ← | C519* (TGC→TGA) | 1.000 | 1.000 | 0.491 | 0.000 | 0.000 |  |
| *ptsP* ← | coding (1526/2247 nt) | 0.000 | 0.000 | 0.226 | 0.000 | 0.000 |  |
| *mutH* → | P67H (CCT→CAT) | 0.000 | 0.147 | 0.000 | 0.000 | 0.000 | methyl‑directed mismatch repair protein |
| *sstT* → | K37N (AAA→AAC) | 0.086 | 0.000 | 0.000 | 0.000 | 0.000 | sodium: serine/threonine symporter |
| *tdcA* ← | G25D (GGC→GAC) | 0.051 | 0.000 | 0.000 | 0.000 | 0.000 | tdc operon transcriptional activator |
| *yhfZ* ← / ← *trpS* | intergenic (‑223/+67) 3,512,567, G→A | 0.000 | 0.000 | 0.121 | 0.000 | 0.000 | putative DNA‑binding transcriptional regulator/tryptophanyl ‑tRNA synthetase |
| *yhfZ* ← / ← *trpS* | intergenic (‑242/+48) 3,512,586, C→A | 0.000 | 0.000 | 0.135 | 0.000 | 0.000 |  |
| *rhsB* → | K1374N (AAG→AAT) | 0.000 | 0.000 | 0.229 | 0.000 | 0.000 | Rhs family putative polymorphic toxin, putative neighboring cell growth inhibitor |
| *rbbA* ← | E123G (GAG→GGG) | 0.096 | 0.000 | 0.000 | 0.000 | 0.000 | ribosome‑associated ATPase: ATP‑binding protein/ATP‑binding membrane protein |
| *bcsC* ← | R546R (AGA→AGG) | 0.076 | 0.000 | 0.000 | 0.000 | 0.000 | cellulose synthase subunit |
| *bcsC* ← | A305A (GCT→GCC) | 0.119 | 0.000 | 0.000 | 0.000 | 0.000 |  |
| *bcsC* ← | V296G (GTG→GGG) | 0.136 | 0.000 | 0.000 | 0.000 | 0.000 |  |
| *trmL* → | L97P (CTG→CCG) | 0.000 | 0.000 | 0.000 | 0.000 | 0.109 | tRNA Leu mC34,mU34 2'‑O‑methyltransferase, SAM‑dependent |
| *waaL* → /  ← *waaU* | intergenic (+15/+17) 3,798,222, A→G | 0.000 | 0.000 | 0.000 | 0.000 | 0.138 | O‑antigen ligase/lipopolysaccharide core biosynthesis |
| *rrsC* → | noncoding (226/1542 nt) 3,942,033, A→G | 0.000 | 0.000 | 0.000 | 0.000 | 1.000 | 16S ribosomal RNA of *rrnC* operon |
| *rrlC* → | noncoding  (2222/2904 nt) 3,945,925, C→G | 0.000 | 0.000 | 0.097 | 0.000 | 0.000 | 23S ribosomal RNA of *rrnC* operon |
| *ilvL* → / → *ilvX* | intergenic (+46/‑41)  3,950,466, G→T | 0.000 | 0.000 | 0.000 | 0.000 | 1.000 | ilvG operon leader peptide/uncharacterized protein |
| *wecA* → | A330V (GCA→GTA) | 0.000 | 0.000 | 0.000 | 0.111 | 0.000 | UDP‑GlcNAc: undecaprenylphosphate GlcNAc‑1‑phosphate transferase |
| *yihL* → | V8I (GTA→ATA) | 0.051 | 0.000 | 0.000 | 0.000 | 0.000 | putative DNA‑binding transcriptional regulator |
| *yihQ* ← | A552E (GCA→GAA) | 0.061 | 0.000 | 0.000 | 0.000 | 0.000 | alpha‑glucosidase |
| *hslU* ← | I343L (ATT→CTT) | 0.115 | 0.000 | 0.000 | 0.000 | 0.000 | molecular chaperone and ATPase component of HslUV protease |
| *rpoB* → | D654Y (GAC→TAC) | 0.000 | 0.000 | 0.000 | 0.377 | 0.000 | RNA polymerase, beta subunit |
| *rpoC* → | R1075C (CGT→TGT) | 0.000 | 0.000 | 0.000 | 0.133 | 0.000 | RNA polymerase, beta prime subunit |
| *thiC* ← | G395D (GGT→GAT) | 0.000 | 0.143 | 0.000 | 0.000 | 0.000 | phosphomethylpyrimidine synthase |
| *pgi* → | V457L (GTG→TTG) | 0.000 | 0.000 | 0.000 | 0.125 | 0.000 | glucosephosphate isomerase |
| *dinF* → | F456S (TTT→TCT) | 0.063 | 0.000 | 0.000 | 0.000 | 0.000 | oxidative stress resistance protein; putative MATE family efflux pump; UV and mitomycin C inducible protein |
| *dnaB* → | A370S (GCA→TCA) | 0.055 | 0.000 | 0.000 | 0.000 | 0.000 | replicative DNA helicase |
| *gltP* → / ← *yjcO* | intergenic (+474/+168) 4,296,268, T→C | 0.253 | 0.000 | 0.000 | 0.000 | 0.000 | glutamate/aspartate:proton symporter/Sel1 family TPR‑like repeat protein |
| *tsaE* → | A39A (GCA→GCG) | 0.053 | 0.000 | 0.000 | 0.000 | 0.000 | tRNA(ANN) t(6)A37 threonylcarbamoyladenosine modification protein; ADP binding protein |
| *valS* ← | T279T (ACT→ACC) | 0.000 | 0.000 | 0.000 | 0.103 | 0.000 | valyl‑tRNA synthetase |
| *fecA* ← | A559T (GCT→ACT) | 1.000 | 1.000 | 0.000 | 0.000 | 0.000 | ferric citrate outer membrane transporter |
| *fecA* ← | G243C (GGC→TGC) | 0.000 | 0.000 | 0.000 | 0.000 | 1.000 |  |
| *fecA* ← | D120Y (GAC→TAC) | 0.000 | 0.000 | 0.327 | 0.000 | 0.000 |  |
| *insA* → | A25A (GCC→GCT) | 0.058 | 0.000 | 0.000 | 0.000 | 0.000 | IS1 repressor TnpA |
| *hsdM* ← | R505G (CGT→GGT) | 0.087 | 0.000 | 0.000 | 0.000 | 0.000 | DNA methyltransferase M |
| **Ancestral** | | | | | | | |
| *[crl]* | 257,908, Δ776 bp | 1.000 | 1.000 | 1.000 | 1.000 | 1.000 | *[crl]* |
| *ychE* → / → *oppA* | intergenic (+254/‑485)  1,299,499, Δ1,199 bp | 1.000 | 1.000 | 1.000 | 1.000 | 1.000 | UPF0056 family inner membrane protein/1 |
| *insB1*–*insA* | 1,978,503, Δ776 bp | 1.000 | 0.000 | 1.000 | 1.000 | 1.000 | *insB1*, *insA* |
| *insA* ← / → *uspC* | intergenic (‑271/‑264)  1,979,486, IS*5* (+) +4 bp | 1.000 | 1.000 | 1.000 | 1.000 | 1.000 | IS1 repressor TnpA/universal stress protein |
| *gatC* ← | pseudogene (1‑2/442 nt)  2,173,361, Δ2 bp | 1.000 | 1.000 | 1.000 | 1.000 | 1.000 | pseudogene, galactitol‑specific enzyme IIC component of PTS; transport; Transport of small molecules: Carbohydrates, organic acids, alcohols; PTS system galactitol‑specific enzyme IIC |
| *[rph]*–*[rph]* | 3,815,859, Δ82 bp | 0.000 | 0.000 | 0.000 | 0.000 | 1.000 | *[rph], [rph]* |
| *glpR* ← / ← *glpR* | intergenic (‑2/+1)  3,560,455:1, +G | 1.000 | 1.000 | 1.000 | 1.000 | 1.000 | pseudogene, DNA‑binding transcriptional repressor; regulator; Energy metabolism, carbon:  Anaerobic respiration; repressor of the glp operon |
| *pyrE* ← / ← *rph* | intergenic (‑41/+25)  3,815,799, A→T | 0.000 | 0.000 | 0.000 | 0.227 | 0.000 | orotate phosphoribosyltransferase/ ribonuclease PH (defective) enzyme; Degradation of RNA; RNase PH |
| *pyrE* ← / ← *rph* | 3,815,809, Δ1 bp | 1.000 | 1.000 | 1.000 | 0.000 | 0.000 |  |
| *ilvG* → | pseudogene (65/663 nt)  3,951,606:1, +C | 1.000 | 1.000 | 0.667 | 0.000 | 0.000 | pseudogene, acetolactate syn. 2 large subunit, valine‑insensitive; acetolactate synthase II, large subunit, cryptic, interrupted |
| *gltP* → / ← *yjcO* | intergenic (+586/+56)  4,296,380:1, +CG | 1.000 | 1.000 | 1.000 | 1.000 | 1.000 | regulator; Energy metabolism, carbon: Anaerobic respiration; repressor of the *glp* operon; glutamate/aspartate: proton symporter/Sel1 family TPR‑like repeat protein |

| **Polymorphisms of control populations at day 200** | | | | | | | |
| --- | --- | --- | --- | --- | --- | --- | --- |
| **Gene** | **Annotation** | **C1** | **C2** | **C3** | **C4** | **C5** | **Description** |
| *rseP* → | I392L (ATA→TTA) | 0.000 | 0.062 | 0.000 | 0.000 | 0.000 | inner membrane zinc RIP metalloprotease; RpoE activator, by degrading RseA; cleaved signal peptide endoprotease |
| *gcd* ← | E145G (GAG→GGG) | 0.000 | 0.000 | 0.081 | 0.000 | 0.000 | glucose dehydrogenase |
| *yaeP* ← / → *yaeQ* | intergenic (‑53/‑113)  214,178, G→C | 0.000 | 0.000 | 0.050 | 0.000 | 0.000 | UPF0253 family protein/PDDEXK superfamily protein |
| *yagH* → | L169P (CTC→CCC) | 0.000 | 0.000 | 0.113 | 0.000 | 0.000 | CP4‑6 prophage; putative xylosidase/arabinosidase |
| *paoD* ← | E48G (GAG→GGG) | 0.108 | 0.000 | 0.000 | 0.000 | 0.000 | moco insertion factor for PaoABC aldehyde oxidoreductase |
| *paoC* ← | E641G (GAG→GGG) | 0.086 | 0.000 | 0.000 | 0.000 | 0.000 | PaoABC aldehyde oxidoreductase, Moco‑containing subunit |
| *mhpT* → | E244G (GAG→GGG) | 0.000 | 0.000 | 0.082 | 0.000 | 0.000 | 3‑hydroxyphenylpropionic transporter |
| *ybaA* → | E77G (GAG→GGG) | 0.000 | 0.058 | 0.000 | 0.000 | 0.000 | DUF1428 family protein |
| *ybcO* → | D62N (GAC→AAC) | 0.000 | 0.066 | 0.000 | 0.000 | 0.000 | DLP12 prophage; uncharacterized protein |
| *rhsC* → | A201G (GCG→GGG) | 0.000 | 0.000 | 0.000 | 0.083 | 0.000 | Rhs family putative polymorphic toxin |
| *sucA* → | E324G (GAG→GGG) | 0.000 | 0.000 | 0.000 | 0.000 | 0.102 | 2‑oxoglutarate decarboxylase, thiamine triphosphate‑binding |
| *glnQ* ← | E200G (GAG→GGG) | 0.069 | 0.000 | 0.000 | 0.000 | 0.000 | glutamine transporter subunit |
| *hyaA* → | A298S (GCG→TCG) | 0.000 | 0.000 | 0.000 | 0.071 | 0.000 | hydrogenase 1, small subunit |
| *ycdU* → / ← *serX* | intergenic (+329/+407)  1,097,158, C→T | 0.000 | 0.106 | 0.000 | 0.000 | 0.000 | putative inner membrane protein/tRNA‑Ser |
| *tonB* → | S31P (TCG→CCG) | 0.000 | 0.000 | 0.000 | 0.000 | 0.121 | membrane spanning protein in TonB‑ExbB‑ExbD transport complex |
| *puuC* → | A207G (GCG→GGG) | 0.052 | 0.000 | 0.000 | 0.000 | 0.000 | gamma‑glutamyl‑gamma‑aminobutyraldehyde dehydrogenase; succinate semialdehyde dehydrogenase |
| *fdnG* → | A115S (GCG→TCG) | 0.128 | 0.000 | 0.000 | 0.000 | 0.000 | formate dehydrogenase‑N, alpha subunit, |
| *lsrR* ← | G152S (GGC→AGC) | 0.000 | 0.000 | 0.060 | 0.000 | 0.000 | lsr operon transcriptional repressor |
| *malX* → | S483T (TCG→ACG) | 0.000 | 0.000 | 0.055 | 0.000 | 0.000 | fused maltose and glucose‑specific PTS enzymes: IIB component, IIC component |
| *ydiJ* ← / → *ydiK* | intergenic (‑142/‑247)  1,768,827, A→G | 0.000 | 0.000 | 0.065 | 0.000 | 0.000 | putative FAD‑linked oxidoreductase/UPF0118 family inner membrane protein |
| *torZ* ← | P251P (CCT→CCA) | 0.000 | 0.000 | 0.050 | 0.000 | 0.000 | trimethylamine N‑oxide reductase system III, |
| *yedJ* ← | E95G (GAG→GGG) | 0.082 | 0.000 | 0.000 | 0.000 | 0.000 | putative HD superfamily phosphohydrolase |
| *wcaC* ← | E186G (GAG→GGG) | 0.000 | 0.135 | 0.000 | 0.000 | 0.000 | putative glycosyl transferase |
| *udk* ← | I190N (ATC→AAC) | 0.000 | 0.000 | 0.094 | 0.000 | 0.000 | uridine/cytidine kinase |
| *tktB* → | S305G (AGC→GGC) | 0.000 | 0.000 | 0.063 | 0.000 | 0.000 | transketolase 2, thiamine triphosphate‑binding |
| *der* ← | E51G (GAG→GGG) | 0.090 | 0.000 | 0.000 | 0.000 | 0.000 | GTPase; multicopy suppressor of ftsJ |
| *iscS* ← | Y177H (TAT→CAT) | 0.000 | 0.055 | 0.000 | 0.000 | 0.000 | cysteine desulfurase (tRNA sulfurtransferase), |
| *hybO* ← | G281G (GGC→GGT) | 0.000 | 0.058 | 0.000 | 0.000 | 0.000 | hydrogenase 2, small subunit |
| *fadH* → | I506V (ATT→GTT) | 0.000 | 0.000 | 0.000 | 0.000 | 0.089 | 2,4‑dienoyl‑CoA reductase, NADH and FMN‑linked |
| *deaD* ← | S223G (AGC→GGC) | 0.000 | 0.000 | 0.000 | 0.000 | 0.052 | ATP‑dependent RNA helicase |
| *glmM* ← | E317G (GAG→GGG) | 0.069 | 0.000 | 0.000 | 0.000 | 0.000 | phosphoglucosamine mutase |
| *yhdW* → | pseudogene (331/960 nt)  3,419,437, C→A | 0.000 | 0.000 | 0.000 | 0.103 | 0.000 | pseudogene, amino‑acid transporter homology; putative transport; Not classified; putative periplasmic binding transport protein; |
| *nirB* → | A343E (GCA→GAA) | 0.000 | 0.235 | 0.000 | 0.000 | 0.108 | nitrite reductase, large subunit, NAD(P)H‑binding |
| *nikC* → | S6P (TCT→CCT) | 0.066 | 0.000 | 0.000 | 0.000 | 0.000 | nickel transporter subunit |
| *rhsB* → | S611S (AGC→AGT) | 0.000 | 0.000 | 0.055 | 0.000 | 0.000 | Rhs family putative polymorphic toxin, putative neighboring cell growth inhibitor |
| *rbbA* ← | S53G (AGC→GGC) | 0.112 | 0.000 | 0.000 | 0.000 | 0.000 | ribosome‑associated ATPase: ATP‑binding protein/ATP‑binding membrane protein |
| *bcsC* ← | A27A (GCT→GCC) | 0.000 | 0.000 | 0.000 | 0.000 | 0.071 | cellulose synthase subunit |
| *dgoR* ← | E47G (GAG→GGG) | 0.000 | 0.000 | 0.080 | 0.000 | 0.000 | putative DNA‑binding transcriptional regulator |
| *recQ* → | G12G (GGA→GGG) | 0.000 | 0.058 | 0.000 | 0.000 | 0.000 | ATP‑dependent DNA helicase |
| *rhaA* ← | S406G (AGC→GGC) | 0.086 | 0.000 | 0.000 | 0.000 | 0.000 | L‑rhamnose isomerase |
| *ftsN* ← | S22P (TCA→CCA) | 0.000 | 0.128 | 0.000 | 0.000 | 0.130 | essential cell division protein |
| *ppc* ← / ← *argE* | intergenic (‑375/+223)  4,153,473, G→T | 0.000 | 0.061 | 0.000 | 0.000 | 0.000 | phosphoenolpyruvate carboxylase/acetylornithine deacetylase |
| *thiC* ← | E169G (GAG→GGG) | 0.110 | 0.000 | 0.000 | 0.000 | 0.000 | phosphomethylpyrimidine synthase |
| *phnO* ← | Y133H (TAC→CAC) | 0.000 | 0.000 | 0.065 | 0.000 | 0.000 | aminoalkylphosphonate N‑acetyltransferase |
| *adiA* ← | A649E (GCA→GAA) | 0.000 | 0.000 | 0.116 | 0.000 | 0.000 | arginine decarboxylase |
| *qorB* ← | V48V (GTG→GTT) | 0.000 | 0.000 | 0.139 | 0.000 | 0.000 | NAD(P)H:quinone oxidoreductase |
| *pyrB* ← | E38G (GAG→GGG) | 0.000 | 0.000 | 0.000 | 0.113 | 0.000 | aspartate carbamoyltransferase, catalytic subunit |
| *yjhF* ← | I176I (ATA→ATT) | 0.000 | 0.000 | 0.000 | 0.000 | 0.058 | putative transporter |
| *yjhG* ← | S40G (AGC→GGC) | 0.080 | 0.000 | 0.000 | 0.000 | 0.000 | putative dehydratase |
| *hsdM* ← | T249T (ACT→ACC) | 0.066 | 0.000 | 0.000 | 0.000 | 0.000 | DNA methyltransferase M |
| *dnaT* ← | E114G (GAG→GGG) | 0.000 | 0.120 | 0.000 | 0.000 | 0.000 | DNA biosynthesis protein (primosomal protein I) |
| *lplA* ← | L166P (CTC→CCC) | 0.000 | 0.000 | 0.000 | 0.000 | 0.101 | lipoate‑protein ligase A |
| **Ancestral** | | | | | | | |
| *[crl]* | 257,908, Δ776 bp | 1.000 | 1.000 | 1.000 | 1.000 | 1.000 | *[crl]* |
| *insB1*–*insA* | 1,978,503, Δ776 bp | 1.000 | 1.000 | 1.000 | 0.000 | 1.000 | *insB1*, *insA* |
| *insA* ← / → *uspC* | intergenic (‑271/‑264)  1,979,486, IS*5* (+) +4 bp | 1.000 | 1.000 | 1.000 | 1.000 | 1.000 | IS1 repressor TnpA/universal stress protein |
| *gatC* ← | pseudogene (1‑2/442 nt)  2,173,361, Δ2 bp | 1.000 | 1.000 | 1.000 | 1.000 | 1.000 | pseudogene, galactitol‑specific enzyme IIC. Transport of small molecules: Carbohydrates, organic acids, alcohols |
| *gltP* → / ← *yjcO* | intergenic (+266/+376)  4,296,060, C→T | 0.053 | 0.116 | 0.129 | 0.000 | 0.069 | glutamate/aspartate: proton symporter/Sel1 family TPR‑like repeat protein |
| *gltP* → / ← *yjcO* | intergenic (+396/+246)  4,296,190, A→G | 0.115 | 0.000 | 0.000 | 0.205 | 0.120 |  |
| *gltP* → / ← *yjcO* | intergenic (+397/+245)  4,296,191, A→C | 0.147 | 0.000 | 0.000 | 0.251 | 0.133 |  |
| *gltP* → / ← *yjcO* | intergenic (+474/+168)  4,296,268, T→C | 0.258 | 0.204 | 0.275 | 0.188 | 0.179 |  |
| *gltP* → / ← *yjcO* | intergenic (+492/+150)  4,296,286, C→T | 0.105 | 0.000 | 0.000 | 0.000 | 0.000 |  |
| *glpR* ← / ← *glpR* | intergenic (‑2/+1)  3,560,455:1, +G | 1.000 | 1.000 | 1.000 | 1.000 | 1.000 | pseudogene, DNA‑binding transcriptional repressor; regulator; Energy metabolism, carbon: Anaerobic respiration; repressor of the *glp* operon |
| *gltP* → / ← *yjcO* | intergenic (+586/+56)  4,296,380:1, +CG | 1.000 | 1.000 | 1.000 | 1.000 | 1.000 | glutamate/aspartate: proton symporter/Sel1 family TPR‑like repeat protein |
