# Supplementary material for: Too much of a good thing: Adaption to iron (II) intoxication in Escherichia coli
Source: Evol Med Public Health. 2021 Jan 18;9(1):53–67. doi: 10.1093/emph/eoaa051 (PMC7937436; doi:10.1093/emph/eoaa051)
Supplement: eoaa051_Supplementary_Data [file eoaa051_supplementary_data.zip › Supplemental_Table_2.docx]

**Supplemental Table 2 – Normalized RNA counts and p-values for all NanoString data.** Gene expression studies were conducted using NanoString technologies on 48 selected genes. Four of the selected genes (yellow) were chosen due to the detection of mutations during DNA sequencing experiments. The remainder of the genes were selected by reviewing the literature for genes that are influence by iron across a subset of metabolic processes including, oxidative stress, biofilm formation, metal and general transport, metabolism, transcription and translation. Here, fold changes in RNA counts between each averaged set of populations for all 48 selected genes are reported, in addition to the two housekeeping genes (blue).

|  | Normalized RNA counts | | |  | Normalized RNA counts | | |  | Normalized RNA counts | | |  | Normalized RNA counts | | |  |
| --- | --- | --- | --- | --- | --- | --- | --- | --- | --- | --- | --- | --- | --- | --- | --- | --- |
| **Gene name** | **Control** | **Control + FeSO_4_** | **Fold Change** | **P-value** | **Fe^2+^** | **Fe^2+^ + FeSO_4_** | **Fold Change** | **P-value** | **Control** | **Fe^2+^** | **Fold Change** | **P-value** | **Control + FeSO_4_** | **Fe^2+^ + FeSO_4_** | **Fold Change** | **P-value** |
| *acnA* | 423.65 | 336.06 | 1.26 | 0.2928 | 2981.89 | 264.74 | 11.26 | 0.0089 | 262.13 | 3764.99 | -14.36 | 0.0048 | 337 | 487.24 | -1.45 | 0.0123 |
| *ariR* | 85.59 | 541.26 | -6.32 | 0.0005 | 397.59 | 650.58 | -1.64 | 0.2765 | 72.92 | 502 | -6.88 | 0.0043 | 542.79 | 1197.37 | -2.21 | 0.0007 |
| *asnS* | 5346.84 | 2527.54 | 2.12 | 0.0147 | 408.22 | 1772.27 | -4.34 | 0.0061 | 1992.45 | 515.43 | 3.87 | 0.0980 | 2534.65 | 3261.79 | -1.29 | 0.2906 |
| *bluR* | 315.68 | 1035.41 | -3.28 | 0.0021 | 177.25 | 575.48 | -3.25 | 0.0001 | 207.16 | 223.8 | -1.08 | 0.6641 | 1038.32 | 1059.14 | -1.02 | 0.9290 |
| *cirA* | 643.4 | 58.83 | 10.94 | 0.0231 | 29.75 | 22.02 | 1.35 | 0.3184 | 366.18 | 37.56 | 9.75 | 0.0060 | 58.99 | 40.53 | 1.46 | 0.0576 |
| *crp* | 13854.57 | 10649.45 | 1.3 | 0.2748 | 3187.77 | 10812.83 | -3.39 | 0.0000 | 4267.6 | 4024.94 | 1.06 | 0.9460 | 10679.4 | 19900.55 | -1.86 | 0.0302 |
| *csgD* | 274.26 | 657.09 | -2.4 | 0.1109 | 199.06 | 167.85 | 1.19 | 0.3646 | 185.12 | 251.34 | -1.36 | 0.4133 | 658.94 | 308.92 | 2.13 | 0.0015 |
| *cyaA* | 1900.85 | 1434.04 | 1.33 | 0.1890 | 694.44 | 765.37 | -1.1 | 0.5688 | 871.1 | 876.81 | -1.01 | 0.9886 | 1438.07 | 1408.63 | 1.02 | 0.9142 |
| *dnaK* | 10501.08 | 1974.03 | 5.32 | 0.0001 | 1978.16 | 1424.77 | 1.39 | 0.2666 | 3418.98 | 2497.65 | 1.37 | 0.7103 | 1979.58 | 2622.22 | -1.32 | 0.1055 |
| *entC* | 875.93 | 282.75 | 3.1 | 0.0995 | 115.67 | 198.61 | -1.72 | 0.0045 | 468.69 | 146.05 | 3.21 | 0.0586 | 283.55 | 365.53 | -1.29 | 0.0002 |
| *fecA* | 25589.96 | 514.36 | 49.75 | 0.0001 | 84.81 | 243.84 | -2.88 | 0.0001 | 6972.12 | 107.08 | 65.11 | 0.0113 | 515.8 | 448.77 | 1.15 | 0.3694 |
| *fur* | 2983.43 | 1355.21 | 2.2 | 0.0056 | 1899.51 | 1623.99 | 1.17 | 0.6173 | 1249.34 | 2398.35 | -1.92 | 0.2981 | 1359.02 | 2988.88 | -2.2 | 0.0054 |
| *gltD* | 12601.48 | 7150.95 | 1.76 | 0.0516 | 439.34 | 4627.06 | -10.53 | 0.0293 | 3955.91 | 554.71 | 7.13 | 0.1029 | 7171.07 | 8515.9 | -1.19 | 0.4369 |
| *ilvG* | 18733.78 | 15821.57 | 1.18 | 0.4353 | 1055.03 | 13467.45 | -12.77 | 0.0034 | 5432.62 | 1332.1 | 4.08 | 0.1966 | 15866.07 | 24786.26 | -1.56 | 0.0714 |
| *iraM* | 70.83 | 188.57 | -2.66 | 0.0200 | 60.47 | 131.13 | -2.17 | 0.0201 | 62.67 | 76.35 | -1.22 | 0.6155 | 189.1 | 241.34 | -1.28 | 0.0293 |
| *kgtP* | 8034.23 | 2523.9 | 3.18 | 0.0075 | 6303.48 | 2892.25 | 2.18 | 0.0042 | 2759.72 | 7958.88 | -2.88 | 0.2062 | 2531 | 5323.07 | -2.1 | 0.0398 |
| *lpxC* | 26301.15 | 11680.36 | 2.25 | 0.0129 | 10492.91 | 14293.18 | -1.36 | 0.0836 | 7126.71 | 13248.53 | -1.86 | 0.5455 | 11713.21 | 26305.98 | -2.25 | 0.0132 |
| *mepM* | 410.62 | 224.35 | 1.83 | 0.0040 | 102.48 | 79.61 | 1.29 | 0.1160 | 255.66 | 129.39 | 1.98 | 0.0046 | 224.98 | 146.52 | 1.54 | 0.0049 |
| *metR* | 284.79 | 300.94 | -1.06 | 0.7737 | 181.99 | 241.12 | -1.32 | 0.1099 | 190.78 | 229.78 | -1.2 | 0.3600 | 301.79 | 443.77 | -1.47 | 0.0029 |
| *mntH* | 1065.4 | 309.58 | 3.44 | 0.1708 | 840.25 | 168.61 | 4.98 | 0.0224 | 548.17 | 1060.92 | -1.94 | 0.4085 | 310.45 | 310.32 | 1 | 0.9919 |
| *murC* | 2291.78 | 1226.79 | 1.87 | 0.0088 | 943.13 | 990.83 | -1.05 | 0.7761 | 1011.69 | 1190.82 | -1.18 | 0.7387 | 1230.24 | 1823.59 | -1.48 | 0.0701 |
| *nudF* | 701.81 | 564.39 | 1.24 | 0.0928 | 352.37 | 404.88 | -1.15 | 0.1894 | 392.54 | 444.91 | -1.13 | 0.5980 | 565.98 | 745.17 | -1.32 | 0.0510 |
| *nusA* | 7450.43 | 3549.6 | 2.1 | 0.0342 | 1259.74 | 3345.56 | -2.66 | 0.0010 | 2598.09 | 1590.57 | 1.63 | 0.5167 | 3559.59 | 6157.36 | -1.73 | 0.0843 |
| *nusG* | 8102.02 | 4858.67 | 1.67 | 0.0901 | 1822.22 | 3631.75 | -1.99 | 0.0214 | 2778.33 | 2300.77 | 1.21 | 0.8080 | 4872.34 | 6684.08 | -1.37 | 0.2173 |
| *ompC* | 20293.65 | 4879.6 | 4.16 | 0.0001 | 9185.76 | 6693.42 | 1.37 | 0.2698 | 5791.59 | 11598.1 | -2 | 0.4861 | 4893.32 | 12318.95 | -2.52 | 0.0004 |
| *ompF* | 94391.43 | 37227.73 | 2.54 | 0.0097 | 10865.32 | 33537.93 | -3.09 | 0.0491 | 19808.67 | 13718.73 | 1.44 | 0.7828 | 37332.44 | 61725.12 | -1.65 | 0.0833 |
| *oxyR* | 4379.43 | 3564.36 | 1.23 | 0.3875 | 884.38 | 2823.71 | -3.19 | 0.0031 | 1698.41 | 1116.63 | 1.52 | 0.5260 | 3574.39 | 5196.92 | -1.45 | 0.1334 |
| *pgaA* | 61.15 | 239.97 | -3.92 | 0.0033 | 67.23 | 86.9 | -1.29 | 0.0907 | 55.72 | 84.88 | -1.52 | 0.2658 | 240.65 | 159.94 | 1.5 | 0.0095 |
| *pgaD* | 44.61 | 172.01 | -3.86 | 0.0032 | 70.3 | 69.41 | 1.01 | 0.9364 | 43.3 | 88.76 | -2.05 | 0.1323 | 172.5 | 127.74 | 1.35 | 0.0319 |
| *ptsP* | 2180.47 | 1604.16 | 1.36 | 0.0876 | 750.22 | 1233.96 | -1.64 | 0.0157 | 972.19 | 947.24 | 1.03 | 0.9567 | 1608.67 | 2271.06 | -1.41 | 0.0683 |
| *rho* | 3811.73 | 2489.58 | 1.53 | 0.0983 | 423.92 | 1518.94 | -3.58 | 0.0037 | 1519.87 | 535.25 | 2.84 | 0.1403 | 2496.58 | 2795.54 | -1.12 | 0.6149 |
| *rpoA* | 54777.77 | 56557.21 | -1.03 | 0.9263 | 1755.95 | 52916.35 | -30.14 | 0.0000 | 12817.19 | 2217.1 | 5.78 | 0.1877 | 56716.28 | 97390.25 | -1.72 | 0.0378 |
| *rpoS* | 13682.39 | 9742.85 | 1.4 | 0.2001 | 19794.2 | 9524.06 | 2.08 | 0.0353 | 4225.12 | 24992.5 | -5.92 | 0.0916 | 9770.25 | 17528.62 | -1.79 | 0.0454 |
| *sodA* | 12252.4 | 3194.76 | 3.84 | 0.0007 | 491.83 | 5017.69 | -10.2 | 0.0003 | 3868 | 620.99 | 6.23 | 0.0795 | 3203.74 | 9234.84 | -2.88 | 0.0030 |
| *sodB* | 2690.52 | 3862.53 | -1.44 | 0.6560 | 18709.35 | 3299.86 | 5.67 | 0.0011 | 1150.21 | 23622.75 | -20.54 | 0.0122 | 3873.4 | 6073.25 | -1.57 | 0.0407 |
| *soxR* | 249.45 | 266.23 | -1.07 | 0.7412 | 315.69 | 143.58 | 2.2 | 0.0159 | 171.59 | 398.6 | -2.32 | 0.0109 | 266.98 | 264.25 | 1.01 | 0.8666 |
| *soxS* | 727.8 | 673.73 | 1.08 | 0.7200 | 1351.62 | 672.61 | 2.01 | 0.0073 | 404.13 | 1706.58 | -4.22 | 0.0023 | 675.63 | 1237.91 | -1.83 | 0.0016 |
| *sufA* | 571.21 | 145.31 | 3.93 | 0.0549 | 1048.41 | 88.03 | 11.91 | 0.0108 | 332.93 | 1323.74 | -3.98 | 0.0812 | 145.72 | 162.02 | -1.11 | 0.4468 |
| *sufB* | 1710.15 | 516.76 | 3.31 | 0.0735 | 2345.31 | 297.79 | 7.88 | 0.0127 | 800.46 | 2961.23 | -3.7 | 0.1033 | 518.21 | 548.07 | -1.06 | 0.5671 |
| *tolC* | 2403.48 | 1117.23 | 2.15 | 0.0058 | 1661.04 | 1218.36 | 1.36 | 0.0599 | 1050.95 | 2097.25 | -2 | 0.2166 | 1120.37 | 2242.34 | -2 | 0.0008 |
| *tufA* | 256355.77 | 81438.54 | 3.15 | 0.0024 | 16644.93 | 91903.04 | -5.52 | 0.0086 | 44053.84 | 21016.17 | 2.1 | 0.6307 | 81667.6 | 169143.58 | -2.07 | 0.0165 |
| *yaaA* | 859.45 | 421.27 | 2.04 | 0.0011 | 232.08 | 302.78 | -1.3 | 0.0581 | 461.62 | 293.03 | 1.58 | 0.1578 | 422.46 | 557.25 | -1.32 | 0.0500 |
| *yggN* | 422.01 | 263.51 | 1.6 | 0.1149 | 172.73 | 230.69 | -1.34 | 0.0454 | 261.32 | 218.09 | 1.2 | 0.4271 | 264.26 | 424.57 | -1.61 | 0.0420 |
| *yghS* | 116.33 | 266.25 | -2.29 | 0.0003 | 77.89 | 127.12 | -1.63 | 0.0022 | 93.21 | 98.35 | -1.06 | 0.7812 | 267 | 233.96 | 1.14 | 0.0780 |
| *yicO* | 65.54 | 241.75 | -3.69 | 0.0008 | 132.5 | 94.49 | 1.4 | 0.1881 | 58.9 | 167.3 | -2.84 | 0.0215 | 242.43 | 173.91 | 1.39 | 0.0498 |
| *ymgA* | 98.1 | 187.02 | -1.91 | 0.0063 | 168.89 | 152.64 | 1.11 | 0.7538 | 81.33 | 213.24 | -2.62 | 0.0286 | 187.55 | 280.93 | -1.5 | 0.0210 |
| *ymgC* | 151.51 | 361.29 | -2.38 | 0.0020 | 164.01 | 325.17 | -1.98 | 0.0261 | 115.15 | 207.09 | -1.8 | 0.0483 | 362.31 | 598.46 | -1.65 | 0.0010 |
| *cysG* | 1167.68 | 1028.69 | 1.14 | 0.4681 | 302.92 | 467.25 | -1.54 | 0.0044 | 589.88 | 382.47 | 1.54 | 0.2487 | 1031.58 | 859.95 | 1.2 | 0.2749 |
| *hcaT* | 247.11 | 251.98 | -1.02 | 0.8119 | 106.51 | 113.19 | -1.06 | 0.4607 | 170.3 | 134.48 | 1.27 | 0.0310 | 252.69 | 208.32 | 1.21 | 0.0221 |
| *idnT* | 251.5 | 246.64 | 1.02 | 0.8119 | 173.23 | 163.01 | 1.06 | 0.4607 | 172.72 | 218.72 | -1.27 | 0.0310 | 247.34 | 300.02 | -1.21 | 0.0221 |
